# GANCIU—Geospatial Analysis with Neural Classification and Image Understanding

**DOI:** 10.3390/jimaging12080382

**Published:** 2026-08-14

**Authors:** Amedeo Ganciu, Giovannangela Ricci, Margherita Solci

**Affiliations:** Department of Architecture, Design and Urban Planning, University of Sassari, 07100 Sassari, Italy; aricci@uniss.it (G.R.); marghe@uniss.it (M.S.)

**Keywords:** land use land cover, OBIA, Random Forest, Mumford–Shah, Ambrosio–Tortorelli, Segment Anything Model, remote sensing, Sardegna

## Abstract

Accurate and up-to-date knowledge of land use and land cover represents one of the central challenges in spatial planning and landscape sciences. In this context, the present work introduces GANCIU (Geospatial Analysis with Neural Classification and Image Understanding), an original hybrid pipeline for the automatic extraction of man-made infrastructure from high-resolution satellite imagery. The primary methodological contribution lies in the sequential integration of four technologically heterogeneous components: a per-pixel Random Forest classifier, a guided image modulation step, edge detection via the Mumford–Shah variational functional solved through the Ambrosio–Tortorelli approximation, and final object delineation via the Segment Anything Model (SAM). Each component does not operate independently but conditions and informs the next: The RF probability map guides the modulation, which in turn directs the sensitivity of the variational step exclusively towards regions of interest; the AT edges provide spatial prompts to SAM, for which its masks are finally filtered by the RF probability in an adaptive manner through a Gaussian Mixture Model. This progressive conditioning scheme constitutes the architectural core of GANCIU and distinguishes it from approaches that combine classification and segmentation in parallel or in purely sequential fashion with each stage conditioning the next but without any reverse correction between them. The Random Forest classifier was trained on 44 manually annotated scenes, geographically disjoint from the twelve independent scenes used for quantitative validation. This validation, based on an instance matching protocol (precision, recall, F1 score, and IoU), confirms the contribution of the full pipeline over a Random-Forest-only baseline: Pooled false positives fall by close to two orders of magnitude (from 8320 to 209), while true positives rise nearly twentyfold (from 5 to 95), with a mean IoU of 0.742 ± 0.060 on correctly matched objects. Notably, the entire pipeline—including SAM-based segmentation—runs end-to-end on a modest, GPU-free consumer laptop (four logical CPU cores, under 16 GB RAM), demonstrating that competitive infrastructure-extraction performance does not require specialised computing hardware.

## 1. Introduction

**Figure 1:**
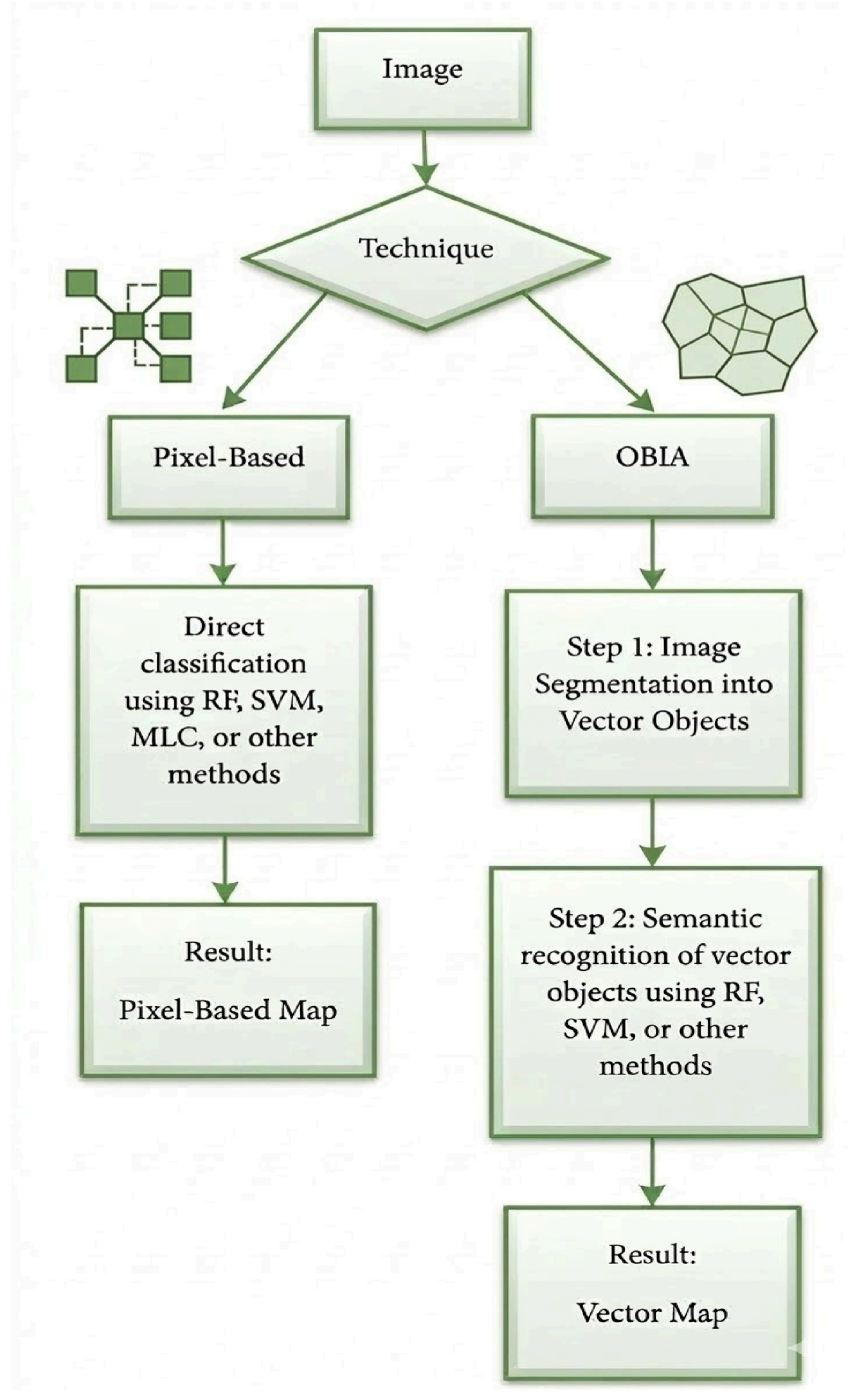
Conceptual diagram comparing the pixel-based and OB IA paradigms.

Accurate and detailed knowledge of land use and land cover (LuLc) is one of the most significant areas of interest in spatial planning and landscape science. It inherently involves diverse research fields, from the strictly ecological to the technological [1,2], and holds central importance in a wide range of studies and applications. These include urban planning, natural and environmental resource monitoring, land-use policy development, and even understanding global climate evolution [3]. Algorithmic approaches to the problem of object recognition in images (trees, rivers, roads, and other features) for LULC purposes can be traced back to two paradigms (Figure 1): The first is structured around the analysis of the spectral signature of each individual pixel; the second, more recent and referred to as Object-Based Image Analysis (OBIA) [4], involves a preliminary segmentation of the image into homogeneous regions (objects) corresponding to real-world entities, distinguishing them from what might be defined as “background” or background noise characterised by the classic “salt-and-pepper” texture [5]; these regions are subsequently subjected to semantic classification based not only on the spectral characteristics of the objects themselves but also on their shape, texture, and spatial context [6].

Comparative studies have demonstrated that OBIA outperforms pixel-based methods in terms of accuracy, particularly when high-resolution imagery is used and complex categories are to be classified, such as those encountered in urban environments [7]. However, this is not a matter of “right or wrong” or “better or worse” but rather of fitness for purpose: The pixel-based approach is simpler, more straightforward, and often sufficient for medium-to-low resolution data where the objective is a general estimate of land cover; in contrast, an OBIA approach is preferable when working with very high resolution imagery, such as that acquired by unmanned aerial vehicles [8] and/or in situations where the classes to be distinguished share similar spectral signatures but differ in shape or texture. Examples include distinguishing a roof from a road [9] or differentiating between tree species, where, in some cases, texture features are more important than purely spectral ones [10].

Classification algorithms can be distinguished into supervised or unsupervised [11] and parametric or non-parametric [12,13]. The first distinction is based on whether a dataset is required to train the classification algorithm. In practice, supervised algorithms are provided with input data alongside the correct corresponding outputs—for instance, “this pixel with these characteristics is grassland.” The algorithm thus learns the relationship between input and output and replicates this procedure across the entire dataset to be analysed. In contrast, unsupervised algorithms do not require a preliminary dataset for training; instead, they autonomously identify patterns, similarities, and clusters within the dataset under analysis [14].

Algorithms can further be distinguished as parametric or non-parametric. The key discriminator here is the requirement for a strong preliminary assumption about the statistical distribution of the data [12], typically assumed to be Gaussian for parametric methods. For non-parametric algorithms, these initial assumptions are unnecessary, making them considerably more adaptable in situations where in-depth prior knowledge of the analysis area is lacking [15]. These categories are not mutually exclusive but rather intersect, providing a deeper interpretative framework for the tools. In this sense, we can identify the following algorithms: supervised parametric, supervised non-parametric, unsupervised parametric, and unsupervised non-parametric (see Table 1).

From the foregoing, and as can be observed in Table 1, there exists a wide variety of technological solutions that differ significantly from one another. However, there is still no single ideal or universally superior algorithm in terms of robustness and result accuracy [16]. A recent review of pixel-level decision fusion strategies for land-cover classification further confirms the centrality of per-pixel classifiers such as RF within current remote sensing practice [17]. A related approach incorporates pixel-level confidence information directly into the fusion process, further improving classification reliability in heterogeneous land-cover contexts [18]. In other words, there is no perfect algorithm, only the one most suited to a specific problem, dataset, user skill level, and final objective, in line with the “No Free Lunch Theorem” formulated by Wolpert in 1995 [19,20]. This principle implies that an algorithm’s superiority only emerges in specific contexts, as its performance results from a trade-off between bias, variance, and computational complexity [21]. Consequently, the relative performance of algorithms is strictly dependent on the nature of the dataset, the size of the training set, and the specific characteristics of the thematic classes to be distinguished [22].

**Figure 2:**
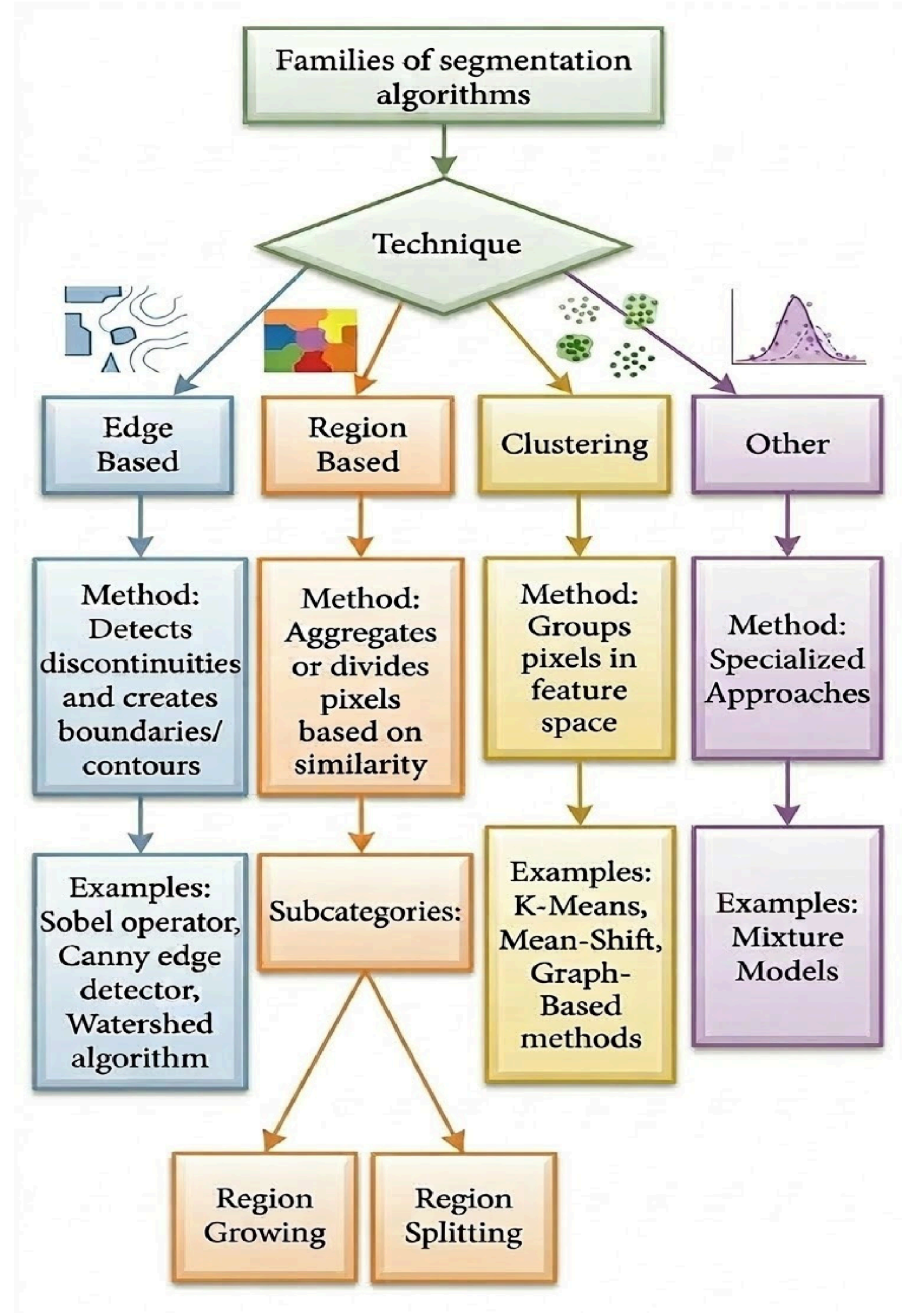
Classification and illustration of image segmentation algorithm families.

A detailed examination of various segmentation algorithms and their applications is provided in the edited volume by Blaschke et al. [6], which compiles several pioneering works in the field. It delves into the theoretical foundations of segmentation and the concept of scale within the principal and most widely used algorithms. A thorough exploration and classification of these would warrant a dedicated discussion; however, a sufficiently general synthesis can be attempted, starting from the seminal and foundational research by Haralick & Shapiro [23], which categorises these algorithms into logical families based on their operating principles (Figure 2).

**Figure 3:**
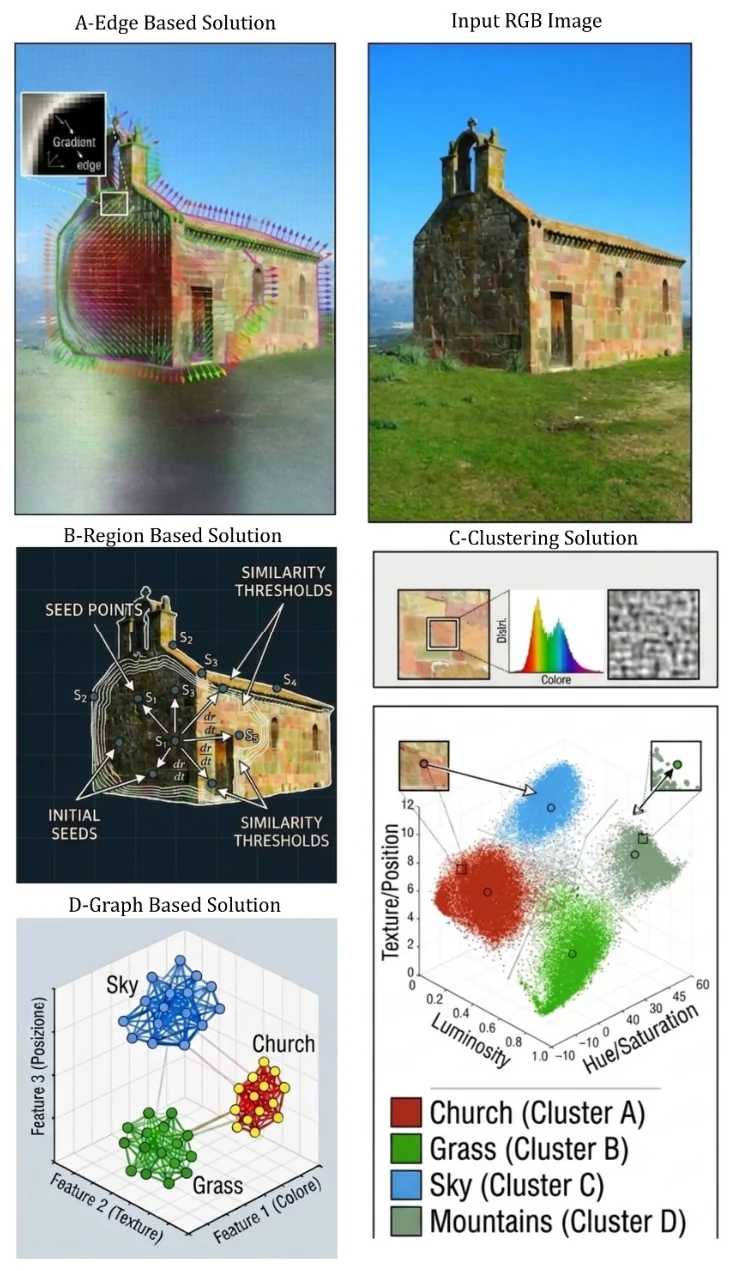
Illustrative examples of the operation of the four families of image segmentation algorithms.

The first family comprises algorithms that operate on the direct detection of edges (edge detection), identifying them where a significant gradient in brightness/colour is measured. For instance, Jin & Davis [24] utilised techniques from this family to identify building outlines using high-resolution imagery, demonstrating the utility of these algorithms within an OBIA workflow for land analysis (Figure 3A).

The second family (region growing) includes algorithms that function in a diametrically opposite manner to the former. Instead of seeking edges, they directly seek homogeneous areas. More specifically, they start from a “seed pixel” and iteratively aggregate adjacent pixels that meet a similarity criterion, for example, a spectral difference below a certain threshold (Figure 3B). A valid and widespread example of this family is the Multiresolution Segmentation developed by Baatz & Schäpe [25], which simultaneously considers spectral and shape similarity.

A third family incorporates clustering techniques that group pixels based on statistical parameters. For example, the K-Means algorithm [26,27], despite its simplicity, remains a benchmark for this technique, although it requires the a priori definition of the number of clusters (K). A more robust and non-parametric evolution is represented by the Mean-Shift algorithm [28], which automatically determines the number of clusters by seeking the modes in the dataset’s density distribution (Figure 3C).

The last category encompasses more modern and specialised algorithms. These develop techniques for modelling the physical properties of the scene, such as illumination or reflectance, to separate objects. Others are based on graph-based approaches (Figure 3D), which represent the image as a graph where nodes are pixels and edges connect neighbours; similarity between pixels is assigned to the edge as a weight attribute, and segmentation occurs by removing edges with minimal weight, thereby partitioning the graph [29,30].

Also, within this last family, one finds techniques based on mixture models, which assume that the data is generated from a mixture of statistical distributions, where each mixture component represents an image region with distinct statistical properties [31].

Of course, from 1985 to the present day, theoretical and technological progression has made significant advances, and the kaleidoscope of algorithms has grown considerably. For example, Pal & Ghosh [32] explicitly acknowledge the role of fuzzy set theory in segmentation; subsequently, Cheng et al. [33] focus on colour image segmentation, incorporating specific methods, such as those based on colour physics, separating reflectance components from illumination to achieve segmentation more robust to changes in light, shadows, and reflections.

Furthermore, the advent of deep learning has revolutionised the playing field, developing methods capable of learning hierarchical features according to neural network architectures, e.g., Fully Convolutional Networks (FCNs) or U-shaped Networks (U-Nets), marking a significant leap forward in performance (Garcia et al. [34]). More recently, dual-path architectures that explicitly reintroduce frequency-domain information alongside spatial features have further advanced deep-learning-based semantic segmentation of remote sensing imagery, achieving state-of-the-art results on standard building-extraction benchmarks such as the ISPRS (International Society for Photogrammetry and Remote Sensing) Vaihingen and Potsdam datasets [35]. For conceptual accuracy, deep learning technology, like Fuzzy logic or Graph Theory itself, could be considered as transversal tools, potentially applicable to all classical segmentation families, rather than as criteria for generating new families. For instance, one could operate with a region-based approach, automated via a convolutional neural network that incorporates Fuzzy logic.

**Figure 4:**
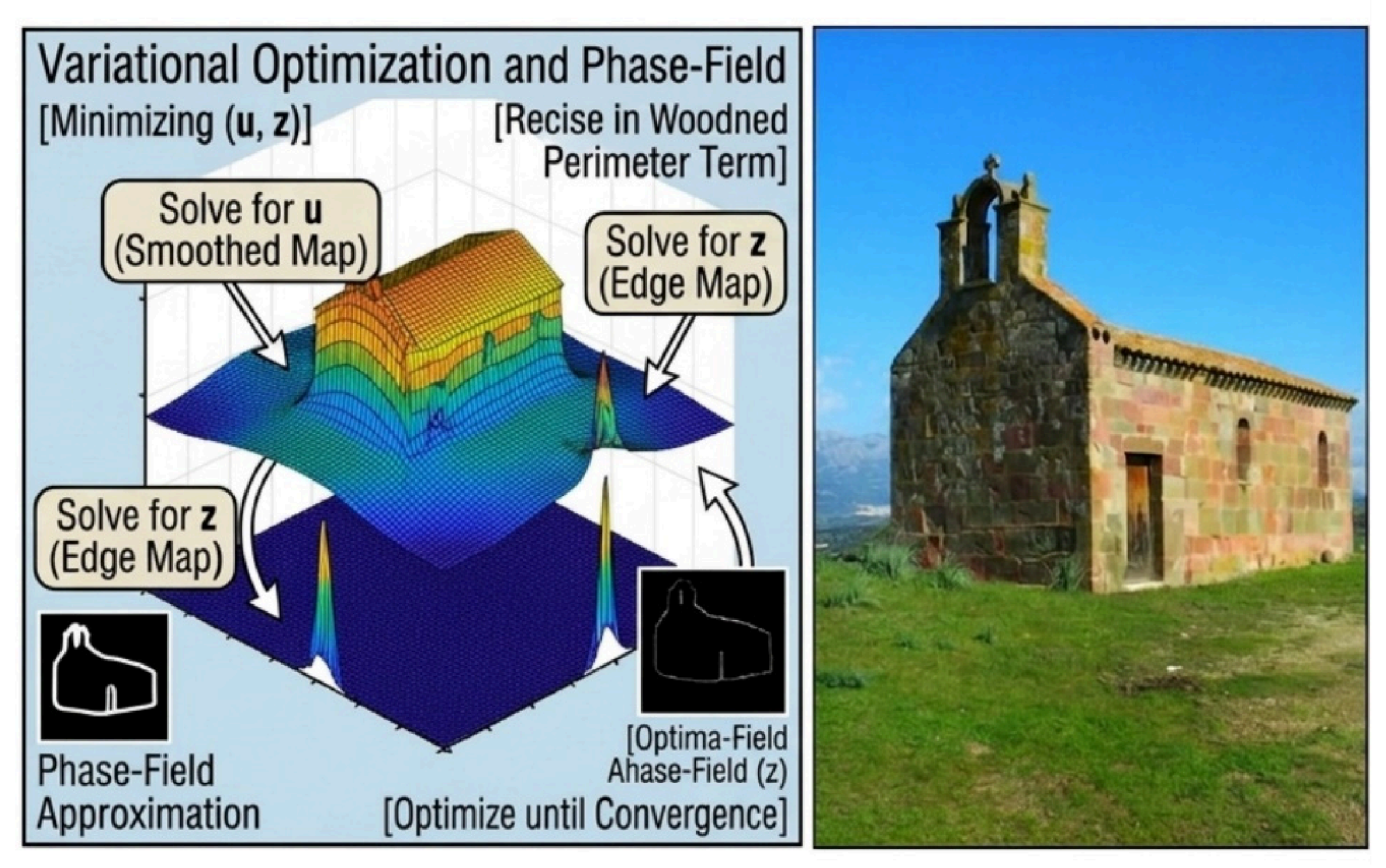
Illustrative example of the segmentation process obtained through the variational method.

An additional class of tools can be identified in variational methods or energy-based models. This class distinguishes itself from the previous ones through its more strictly mathematical and geometric approach to the segmentation problem. Its seminal representative can be found in the Mumford–Shah functional [36]. Through energy optimisation, it allows the image to be segmented into homogeneous regions by differentiating its pixels into those belonging to a “smooth” zone, such as the interior of a sought-after object or the image background, and those identifying the separation, or more precisely, the boundary between objects and background. The technique of image segmentation via this procedure occurs by minimising the energy value produced by the pixels of an image (Figure 4). However, minimising the Mumford–Shah functional directly is highly complex because the set of object boundaries can have wide variability. Minimising the functional means finding the best segmentation of an image that balances three objectives [37]:

- Having smooth regions, inside of which there are no large variations in the image; thus, low variance between pixel values.
- Having boundaries that are as short and regular as possible.
- The processed image, where pixels are classified as boundary or background, must resemble the original image as closely as possible.

In more formal terms, for an original two-dimensional image g(x,y), one seeks an image u(x,y) that simplifies g(x,y) and a set of boundaries K, which minimises the overall energy E(u,K) as in the following (Equation (1)):

(1) $$E(u,K)=\;\int_{\Omega \backslash K}{\left|\nabla u\right|}^{2}dxdy+\;\mu \int_{\Omega }{\left(u-g\right)}^{2}dxdy+v*length\;\left(K\right)$$

The first part of the equation, known as the regularity term, “penalises” variations within the smooth regions. In other words, the simplified image u(x,y) should be as uniform as possible inside each region, such as the interior of an object or the background itself.

The second term controls the fidelity or, more precisely, the similarity between the original image g(x,y) and its simplified copy u(x,y) through the tuning parameter μ. Assuming a high value for this parameter forces the objects in the copy u(x,y) to be characterised by a high level of detail. However, this inevitably leads to an increase in the length of the segmentation boundary K and, consequently, in the overall energy value E(u,K).

Finally, the last part of the functional penalises overly long and complex boundaries through the parameter ν. Increasing this parameter produces solutions with shorter and simpler contours [38], but conversely, there is a risk of losing useful details that are not merely “noise.”

The original formulation of the problem by Mumford and Shah stipulates that K must be a finite union of closed, regular (more precisely, analytic) curves. However, as is customary in modern mathematical analysis, it is preferable to work with a version where such regularity is not directly required. One can then attempt to prove that the minimising sets K are, in fact, more regular, using their minimality to demonstrate this. For generic sets K, the concept of length can be generalised using the one-dimensional Hausdorff measure. With this nuance, for each fixed K, the functional E(u,K) can be minimised with respect to the variable u in a classical manner, using appropriate Sobolev spaces, to obtain minimisers, $u_{K}$. The subsequent minimisation—that is, minimising $E(u_{K},K)$ with respect to K—is more complex and requires considering the Hausdorff convergence of sets K and the minimality properties of the solution [39]. Solving minimisation problems involving the Mumford–Shah functional in the presented form is therefore extremely complex, as it involves two variables, the function u and the set K, of very different natures.

A completely different viewpoint is due to De Giorgi [40,41], who interprets the variable u as the sole variable of the problem, with the set K replaced by the set S(u) of essential discontinuity points of u. To implement this approach, appropriate spaces of special functions of bounded variation (from which the acronym SBV is derived) were introduced and studied. Using these, the functional can be defined as follows (Equation (2)):

(2) $$F\left(u\right)=F(u,\;S\left(u\right))$$

This represents the weak form of the Mumford–Shah functional, where K is replaced by S(u) and a suitable meaning is given to the “approximate gradient,” allowing for the resolution of related minimisation problems. Furthermore, regularity theorems then ensure that S(u) can be considered as a union of curves [36,37]. Despite this conceptual effort, the implementation of a direct numerical minimisation of the Mumford–Shah functional—even via the De Giorgi [40,41] method—still presents significant difficulties. These stem from its dependence on the unknown one-dimensional set K, whether considered as an arbitrary closed set or as a finite union of closed curves. However, the formulation in terms of SBV functions allows the functional F(u) to be framed within a variational setting. In this setting, one can construct approximations capable of guaranteeing the convergence of minimisation problems, understood as the convergence of both the minimal values and the minimising functions. A sequence of functionals possessing this property is said to Gamma-converge to F [42]. This approach offers greater flexibility, which translates into the possibility of developing approximations in spaces different from that of the Gamma limit—in our case, the Mumford–Shah functional in its weak form [43].

The GANCIU (Geospatial Analysis with Neural Classification and Image Understanding) pipeline implements a four-step workflow that combines a supervised Random Forest (RF) classifier, the Mumford–Shah functional minimised through the Ambrosio–Tortorelli (AT) approximation, and the Segment Anything Model (SAM) to extract and delineate man-made infrastructure objects from very-high-resolution satellite imagery. The four steps are executed in sequence: (1) per-pixel probabilistic classification via RF, (2) image modulation guided by the RF probability map, (3) edge detection via AT applied to the modulated image, and (4) object delineation via SAM conditioned on the AT edge map, with final selection based on RF probability. The main contributions of this work are threefold. First, GANCIU integrates four methodologically heterogeneous components: a per-pixel classifier, a variational PDE-based edge model, and a promptable foundation segmentation model into a single, progressively conditioned pipeline, in contrast to the prevailing trend toward monolithic end-to-end deep learning architectures for infrastructure extraction. Second, the RF-probability-guided image modulation step introduced between classification and edge detection is, to the authors’ knowledge, a novel mechanism for suppressing background clutter ahead of variational edge detection, rather than relying on the edge detector alone to discriminate infrastructure from background texture. Third, this study contributes a rigorous, instance-level quantitative validation protocol (Hungarian-algorithm-based matching and precision/recall/F1 score/IoU with mean ± standard deviation) over twelve geographically disjoint scenes and a Random-Forest-only ablation baseline, together with full sensor/date/resolution traceability, addressing a methodological gap common to many hybrid remote sensing pipelines.

## 2. Methods

The following subsections describe each step in detail, including the mathematical formulation, the parameter calibration strategy, and the computational implementation.

### 2.1. Per-Pixel Classification with Random Forest

The first step produces a continuous probability map *P*(*x*,*y*) ∈ [0, 1] over the input image, where values close to 1 indicate pixels likely belonging to an infrastructure object (building, road, or other man-made structure) and values close to 0 indicate background (vegetation, bare soil, and water). This is accomplished by a Random Forest classifier [44] trained on a set of manually annotated image/ground-truth pairs. The Random Forest (RF) classifier, an ensemble method that generates multiple decision trees from randomly selected subsets of training samples and features [44,45], has established itself as one of the most effective and widely used algorithms for image analysis from satellite data. Its robustness to overfitting, its capacity to handle high-dimensional feature spaces, and its relative insensitivity to noisy input data make it particularly well suited to the spectral and textural complexity of high-resolution imagery [45]. Recent hybrid pipelines combining deep-learning-based feature extraction with RF as the final classifier have confirmed RF’s superior segmentation accuracy over several alternative machine learning classifiers on comparable high-resolution remote sensing imagery [46].

In the context of the GANCIU pipeline, the RF classifier is applied at the pixel level to a set of 45 features per pixel, including multi-scale texture descriptors derived from the Gray-Level Co-Occurrence Matrix (GLCM) [47], Local Binary Patterns (LBPs) [48], and Gabor filters [49] at four frequencies and four orientations, alongside chromatic and gradient features. The resulting per-pixel probability map guides the subsequent segmentation stage, acting as a spatial prior that directs the variational algorithm towards regions of genuine interest and away from spectrally homogeneous background areas.

The features are designed to be invariant to absolute reflectance levels and, therefore, to seasonal variation, atmospheric correction differences, and illumination changes by relying exclusively on local statistics and relative spectral relationships. The feature set consists of four groups:

- Gray-Level Co-Occurrence Matrix (GLCM) features [47] encode the spatial co-occurrence statistics of pixel intensity values. For each of four window sizes (3, 7, 15, and 31 pixels), four Haralick descriptors are computed: contrast, homogeneity, energy, and correlation. This yields 16 features capturing texture at multiple spatial scales, from sub-metric grain to building-block level.
- Local Binary Pattern (LBP) [48] features encode the local micro-texture around each pixel by comparing it to its neighbours on a ring of radius 2 (16 sampling points, uniform patterns). Three statistical summaries—entropy, energy, and uniformity—are computed independently on each of the three Red-Green-Blue channels (RGB), yielding 9 features. The per-channel computation preserves chromatic texture information that would be lost by grayscale conversion.
- The Gabor filter bank [49] at 4 frequencies (0.05, 0.15, 0.30, and 0.45 cycles/pixel) and 4 orientations (0°, 45°, 90°, and 135°) captures directional frequency content characteristics of structured surfaces such as rooftops and road surfaces. The mean filter response energy is computed for each of the 16 filter combinations, yielding 16 features.
- Chromatic and gradient features, two relative colour ratios (R/G and the variance of the normalised RGB vector), encode spectral properties without dependence on absolute brightness. Two gradient features (gradient magnitude and the ratio of horizontal to vertical gradient) encode local structural orientations. These four features complete the 45-dimensional vector.

The RF model is trained using labelled pixel pairs extracted from a set of manually annotated scenes. For each training scene, positive samples are drawn from ground-truth infrastructure regions and negative samples from background regions, with a background-to-object ratio of *k* = 6 applied within scenes containing at least one object pixel. Scenes with no object pixels in the ground truth (a genuine category among the training scenes, corresponding to purely agricultural areas with no infrastructure) contribute background samples only without this ratio constraint, since no per-scene object count exists to which it could be applied. The pooled training set, therefore, reflects both populations: object-containing scenes sampled near the nominal 6:1 target, and object-free scenes contributing background exclusively, yielding an overall background-to-object ratio of approximately 1:18.5 (Table 2). The ensemble comprises 300 decision trees; the probability output for the positive class (infrastructure) is the fraction of trees that vote for that class.

At inference time, the full input image is processed in non-overlapping tiles of 1000 × 1500 pixels with a 64-pixel overlap border. The overlap border is used for feature extraction (to avoid boundary artefacts in texture filters with large kernels) but discarded before assembling the probability map. For a typical image of 6000 × 4000 pixels, this tile strategy substantially reduces peak memory consumption relative to processing the full image at once, enabling execution on memory-constrained hardware without GPU acceleration. After assembling the full probability map, a binarisation threshold is computed adaptively from the distribution of *P*(*x*,*y*) using Otsu’s method [50]. If the Otsu quality criterion is η < 0.5 (indicating a flat, poorly bimodal distribution), the threshold is re-estimated using a two-component Gaussian Mixture Model (GMM) [51], taking the intersection of the two fitted Gaussians as the decision boundary.

The class imbalance in the training set (916,662 object samples versus 16,933,338 background samples, roughly 1:18.5) reflects the natural scarcity of infrastructure pixels in rural imagery and motivated the balanced class weighting used during training. The resulting precision–recall asymmetry on the test set—near-perfect background classification (*p* = 0.999, R = 0.962) against markedly lower object precision but very high object recall (*p* = 0.581, R = 0.980)—is a deliberate consequence of this weighting: RF is tuned to miss as few true objects as possible, at the cost of many false positives, which the downstream AT, SAM, and GMM stages are then responsible for filtering out. The OOB score (0.9627) confirms the strong internal consistency of the trained ensemble (Table 3).

**Figure 5:**
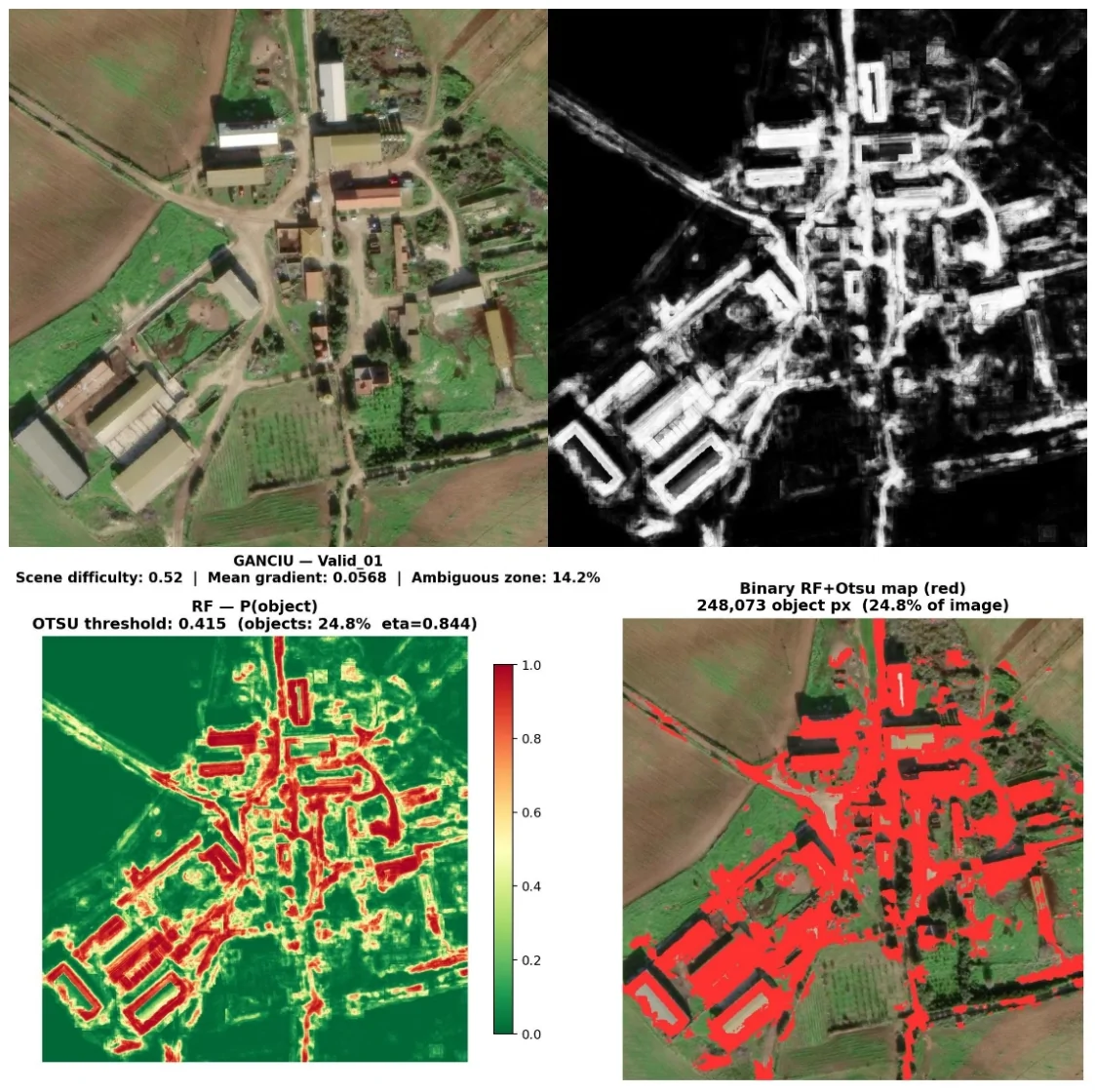
Step 1’s output for validation scene Valid_01: original RGB (**top-left**); grayscale rendering (**top-right**); RF probability map P(object) with adaptive Otsu threshold = 0.415 and η = 0.844 (**bottom-left**); and resulting binary classification mask overlaid in red, 248,073 object pixels—24.8% of the image (**bottom-right**).

Two files are produced by this step: (i) *_probabilita.tif, (Figure 5) the continuous probability map in float32 format; and (ii) *_analisi.json, a JavaScript Object Notation file (JSON) containing the statistical description of the probability distribution in the background, object, and ambiguous zones, including the optimal neutral colour values used in the following step.

### 2.2. Image Modulation

The second step produces a modulated version of the input image that suppresses spectral variability in background regions while preserving it in infrastructure regions. This modulated image is the input to the AT functional in the next step. The motivation is to prevent the variational algorithm from detecting edges in low-probability zones (vegetation texture, crop boundaries, and shadow edges), directing its sensitivity exclusively towards regions indicated by the RF classifier as likely infrastructure. For each pixel (*x*, *y*) and each colour channel *c* ∈ {R, G, B}, the modulated image value is computed as follows:

$$I_{c}^{mod}\left(x,y\right)=\;I_{c}\left(x,y\right)*P{\left(x,y\right)}^{\gamma }+n_{c}*\left(1-P{\left(x,y\right)}^{\gamma }\right)$$

where $I_{c}\left(x,y\right)$ is the original pixel value in channel *c*; $P{\left(x,y\right)}^{\gamma }$ is the RF probability map; $n_{c}$ is the neutral value for channel *c*; and *γ* is a user-controlled modulation curve parameter (default γ = 1.0, corresponding to a linear transition). The neutral value $n_{c}$ is determined adaptively for each image from the statistical analysis produced in Step 1: it is chosen as the median (or mode, if the distribution is strongly skewed) of pixel values in channel *c* within the background zone $P{\left(x,y\right)}^{\gamma }<t$, where *t* is the Otsu threshold. This ensures that background pixels converge to a radiometrically appropriate flat colour rather than an arbitrary constant. The parameter *γ* controls the sharpness of the transition between preserved and suppressed regions:

- *γ* = 1.0 (Default): linear transition—a pixel with *p* = 0.5 receives equal weight from original and neutral.
- *γ* < 1.0 (e.g., 0.5): soft transition—more of the original signal is preserved at intermediate probabilities.
- *γ* > 1.0 (e.g., 2.0): hard transition—intermediate probabilities are pushed strongly towards the neutral value

The modulated image is saved as *_modulata.tif (uint8, 3 bands).

Statistics (mean, standard deviation, 75th and 90th percentiles) are extracted from pixels within the high-probability RF zone *P*(*x*,*y*) > *t* so that gradient statistics refer to genuine infrastructure edges rather than background noise. The three AT parameters are then set as follows:

- *μ* = clip (0.05/*ḡ*, 0.1, 10.0), where *ḡ* is the mean gradient magnitude in the RF zone. This scales the data-fidelity term relative to the actual contrast present in the scene.
- *ν* = *μ*/1000. The contour-length penalty is kept proportional to *μ* to maintain a consistent balance between regularisation and fidelity across scenes.
- ε Schedule: a decreasing sequence of ε values is selected from three pre-defined schedules (short, medium, and long) based on the scene’s difficulty index—defined as the fraction of ambiguous pixels relative to the fraction of RF-positive pixels. Harder scenes use longer schedules that start from larger ε values (e.g., [4.0, 2.0, 1.0, 0.5, 0.2, 0.1, 0.05, 0.02, 0.01]) to ensure convergence. This progressively decreasing ε schedule follows established practice for the numerical selection of the regularisation parameter in the Ambrosio–Tortorelli approximation [52].

The calibrated parameters are stored in *_parametri_AT_su_modula.json and read automatically by the next step.

### 2.3. Edge Detection via Ambrosio–Tortorelli

The Ambrosio–Tortorelli approximation was selected over fractional calculus-based edge operators or a learned (deep learning) edge detector for four reasons. First, it rests on a well-established theoretical foundation, “Γ” convergence to the Mumford–Shah functional, whereas fractional calculus edge operators are comparatively heuristic for the specific piecewise smooth, free discontinuity decomposition targeted here. Second, it requires no labelled training data, consistent with the pipeline’s broader design goal of minimising supervised deep learning requirements relative to end-to-end alternatives. Third, it is formulated as a continuous variational problem solved directly on a continuous-valued image, a natural mathematical fit with the RF probability-guided modulation step that precedes it in Section 2.2; a discrete or learned edge detector would require a different interface at this point in the pipeline. Fourth, it carries no GPU dependency at this stage, keeping the computational profile of the pipeline lightweight outside of the SAM step.

The third step applies the Ambrosio–Tortorelli (AT) approximation of the Mumford–Shah functional to the modulated image, producing a continuous edge map *v*(*x*,*y*) ∈ [0, 1] in which low values identify strong discontinuities (edges) and values close to 1 correspond to smooth interior regions. Among the various solutions for numerically approximating the Mumford–Shah functional, the one proposed and studied by Ambrosio and Tortorelli has been particularly widely used, especially in computational contexts [53]. The core idea behind this approximation is the use of an additional variable or parameter: a function v, such that 1−v is a “regularised” version of the characteristic function of the discontinuity set S(u). In essence, the function v takes the value 1 “far” from the discontinuity set of u and 0 “close” to it. The “regularized” term is as follows:

$$\int_{\Omega }v^{2}{\left|\nabla u\right|}^{2}dxdy$$

It is thus well-defined even if the function *u* is discontinuous on *S*(*u*), meaning that, over this set, the product with *v*^2^ is null. The term involving the length of *S*(*u*) is replaced with a continuous approximation dependent on the parameter *n*, with *n* tending to infinity:

$$\int_{\Omega }\left(n{\left(v-1\right)}^{2}+\;\frac{1}{n}{\left|\nabla v\right|}^{2}\right)dxdy$$

For large *n*, the finiteness of the first term in this integral implies that *v* is close to the constant 1 except on a set of small measures. Recalling that the value of *v* is 0 on *S*(*u*), this means the function *v* must undergo a transition from 0 to 1 in a neighbourhood of *S*(*u*). This transition, however, is penalised by the second integral. As *n* tends to infinity, these two terms balance each other, and their combined value tends to a multiple of the length of *S*(*u*) due to a Gamma-convergence result related to phase transitions [54].

The Ambrosio–Tortorelli functional, dependent on the parameter *n*, thus takes the following overall form:

$$\int_{\Omega }v^{2}{\left|\nabla u\right|}^{2}dxdy+\;\mu \int_{\Omega }{\left(u-g\right)}^{2}dxdy+v\int_{\Omega }\left(n{\left(v-1\right)}^{2}+\frac{1}{n}{\left|\nabla v\right|}^{2}\right)$$

**Figure 6:**
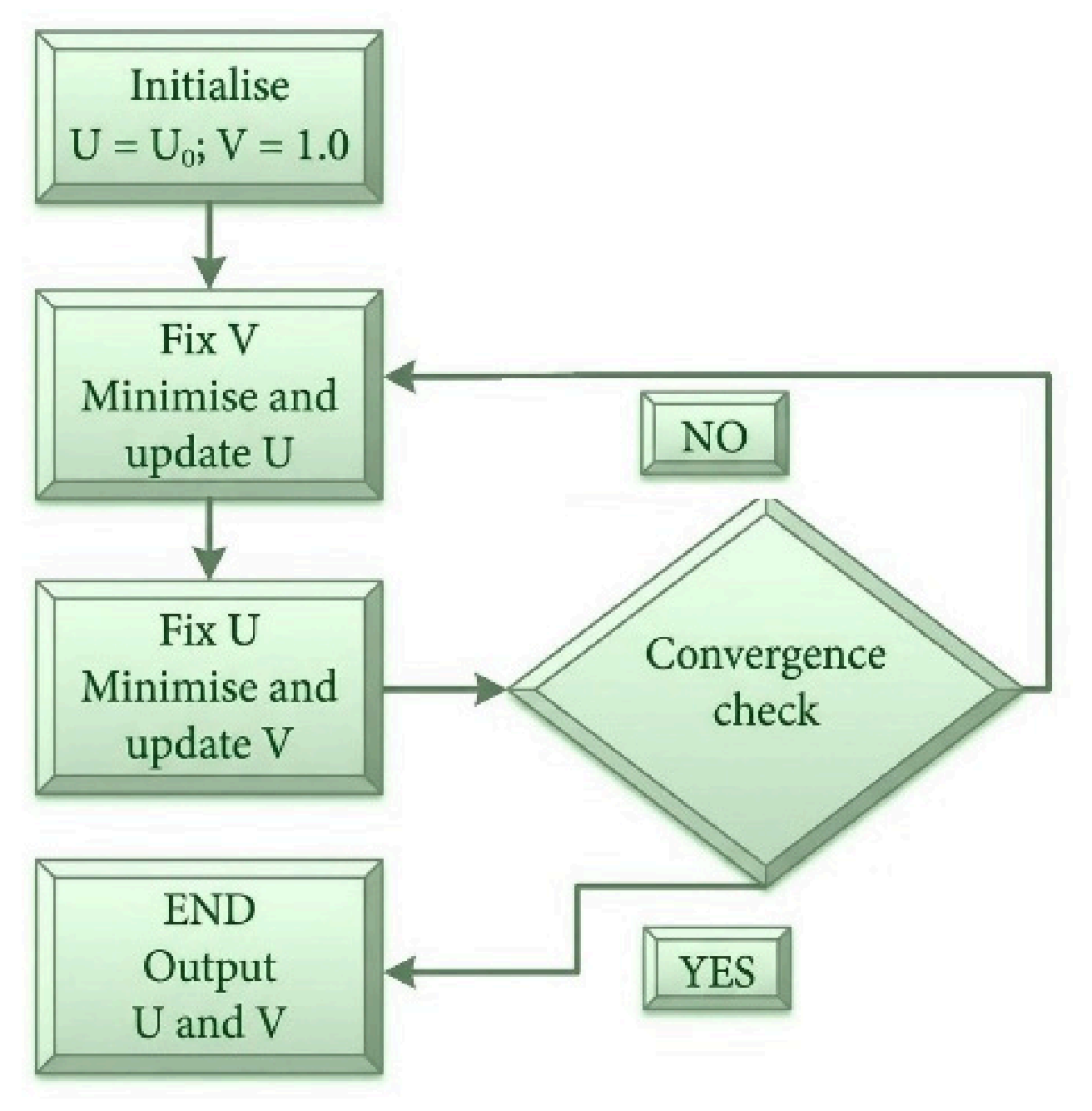
Ambrosio–Tortorelli execution algorithm.

Following the solution proposed by Ambrosio and Tortorelli, we must find the function u (the approximated image) and the function v (the edge map) that minimise the value of E(u,v). The numerical implementation follows these logical steps (Figure 6):

(a) Initialisation:
  - A matrix *u*(*x*,*y*) is created to approximate the original image *g*(*x*,*y*) as closely as possible. The ideal initial solution is therefore to initialise *u*(*x*,*y*) with the exact numerical values of *g*(*x*,*y*).
  - For the function *v*, which will contain the edge map, a matrix of the same dimensions as *u*(*x*,*y*) is initialised, with all its elements assigned the value *v* = 1. Starting with the value 1 everywhere means assuming, in the initial phase, that there are no edges in the image—a neutral starting hypothesis. In subsequent iterations, the algorithm will update this matrix, pushing values towards the threshold 0 to identify discontinuities (i.e., the edges of the sought objects).
(b) Iterative Minimisation: Once the matrix v is initialised, the functional is minimised through an iterative process:
  - Minimise with Respect to *u*: The values in the matrix *u*(*x*,*y*) are updated, using the current *v* matrix (initially all 1 s).
  - Minimise with Respect to *v*: Using the newly computed *u*(*x*,*y*) matrix from the previous step, the functional is minimised again, this time with respect to *v*. This yields an updated edge map matrix *v*.
(c) Convergence: These alternating minimisations between the u and v matrices are repeated until the changes in the respective updated matrices are deemed negligible.

Specifically, the minimisation of *u*(*x*,*y*) with respect to *v* is implemented via the Euler–Lagrange equation for *u* (in discrete form), which yields a linear system whereby each element of the matrix *u*(*x*,*y*) is updated as a function of its neighbours. For each pixel, a new value is then computed, balancing the original datum and the regularisation term:

$$u_{new(i,j)}\approx \frac{u_{0}\left(x,y\right)+\frac{\mu }{h^{2}}\sum_{neighbours}v_{neighbours}^{2}*u_{neighbours}^{2}}{1+\frac{\mu }{h^{2}}\sum v_{neighbours}^{2}}$$

where *h* is the spatial step (*h* = 1), and in the case of the first iteration where *v* = 1 everywhere, the expression simplifies to an average between the datum and the mean of its neighbours. Conversely, for the minimisation of *v* with respect to the updated *u*(*x*,*y*), the formula derived from the per-pixel minimisation of the matrix is used:

$$v_{new}\left(x,y\right)\approx \frac{1}{1+K*{\left|\nabla u\left(x,y\right)\right|}^{2}}$$

where the squared magnitude ∣∇*u*∣^2^ is obtained using the centred finite-difference scheme to compute the partial derivatives:

$$\begin{array}{c} {\left|\nabla u\right|}^{2}={\left(\frac{\partial u}{\partial x}\right)}^{2}+{\left(\frac{\partial u}{\partial y}\right)}^{2}\\ \frac{\partial u}{\partial x}\approx \frac{u\left(x,y+1\right)-u\left(x,y-1\right)}{2};\;\frac{\partial u}{\partial y}\approx \frac{u\left(x+1,y\right)-u\left(x-1,y\right)}{2} \end{array}$$

**Figure 7:**
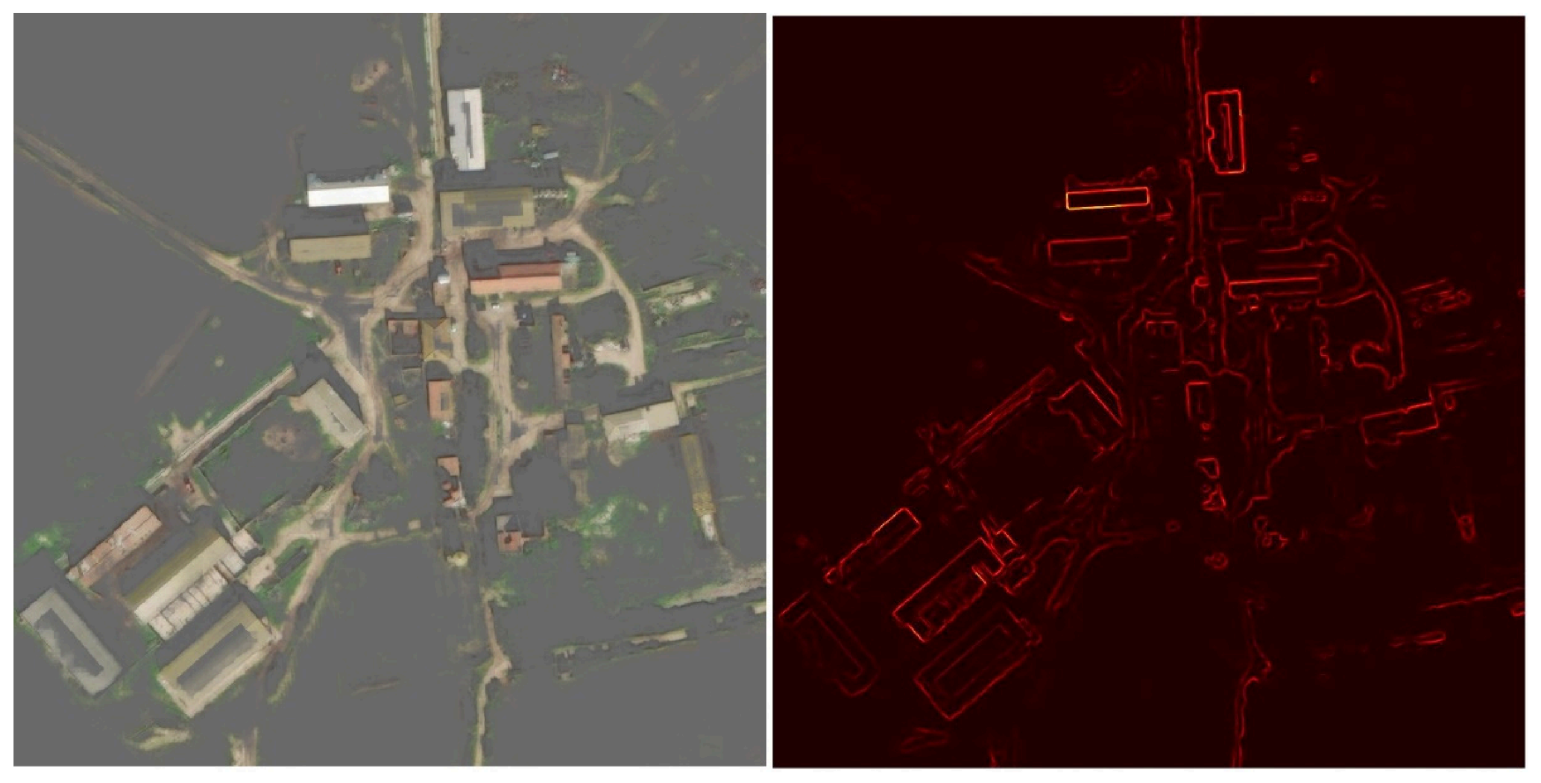
Steps 2–3’s output for validation scene Valid_01: RF-modulated image (**left**), with background regions flattened toward the neutral colour and infrastructure regions retaining original contrast; resulting Ambrosio–Tortorelli edge map (1 − *v*) (**right**), highlighting building and road boundaries.

It should be recalled that, in regions where the gradient is high, *v* becomes small (<<1), allowing *u*(*x*,*y*) to approach the original data more closely in subsequent iterations. Direct application of the AT algorithm to full-resolution images (6000 × 4000 pixels or larger) is computationally intractable on standard hardware. The image is therefore partitioned into tiles of 1000 × 1500 pixels with an overlap border of 128 pixels. Each tile is processed independently; in the overlap zones, results from adjacent tiles are blended using a linearly increasing weight ramp (from 0 at the tile edge to 1 at the interior boundary). This weighted blending prevents discontinuities in the edge map at tile boundaries, which would otherwise produce artefacts in the subsequent SAM step. The output of this step is *_modulata_v_map.tif: a float32 raster with values in [0, 1] (Figure 7).

### 2.4. Object Delineation via SAM

A further contribution to the object delineation stage comes from the Segment Anything Model (SAM) [53], a foundation model developed by Meta AI and trained on over one billion segmentation masks. SAM operates through a promptable architecture: Given a point, bounding box, or other spatial cue, it generates a precise segmentation mask for the corresponding object with zero-shot generalisation across image types and domains. Its application to remote sensing imagery has been systematically evaluated, demonstrating strong performance in delineating buildings, roads, and infrastructure from aerial and satellite data, particularly when combined with an upstream classifier providing candidate object locations [55]. In the workflow presented here, SAM receives point prompts derived from the density peaks of the AT edge map and produces individual object masks that are subsequently filtered by the RF probability map, retaining only objects with interior pixels that exhibit high classification confidence. Beyond the present guided-prompting strategy, other recent efforts to adapt SAM specifically to geographical mobility infrastructure include decoder fine-tuning driven by multimodal prompts [56] and zero-shot workflows built around task-specific data representations [57], illustrating complementary routes toward improving SAM’s applicability to this domain without necessarily retraining the model itself.

**Figure 8:**
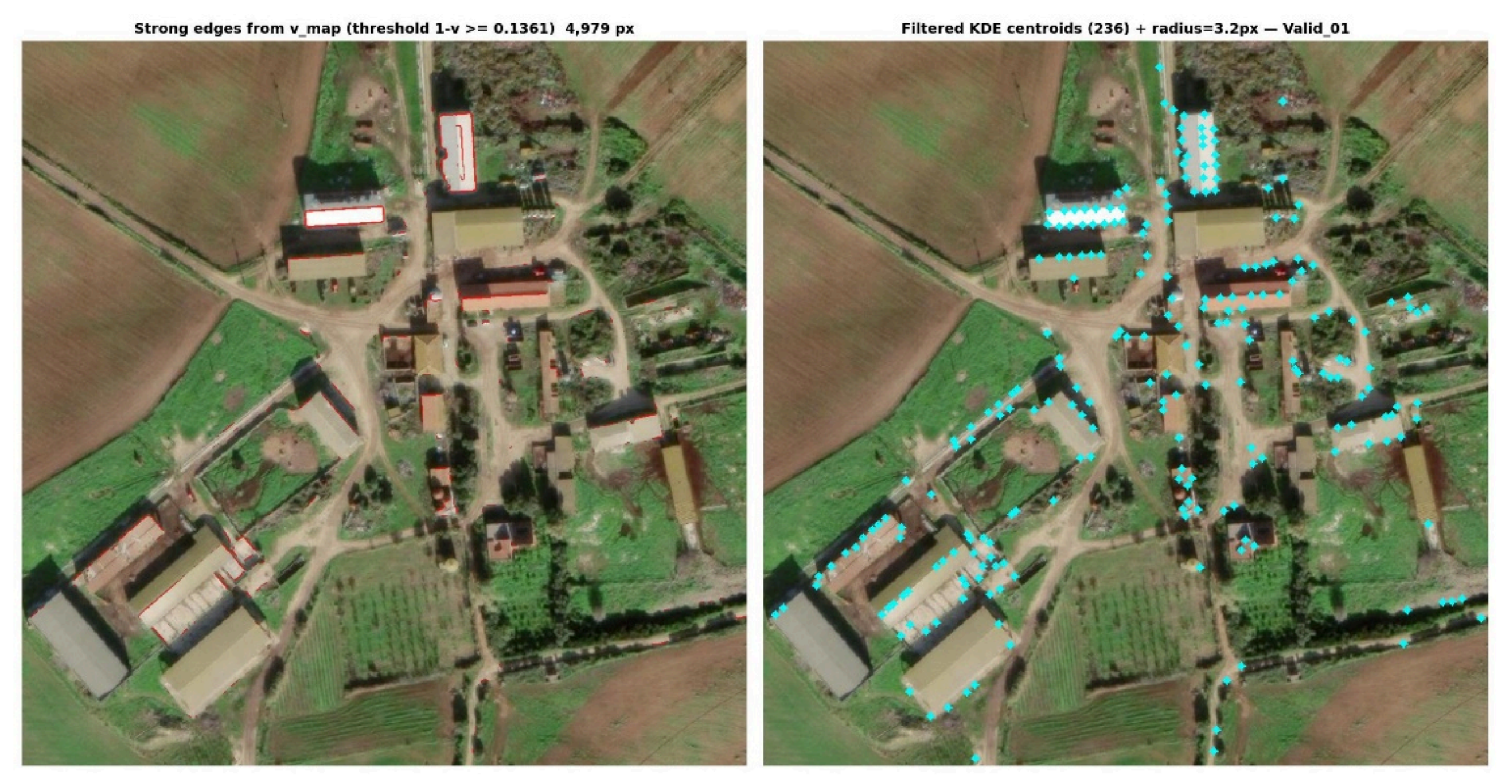
Step 4’s output for validation scene Valid_01: strong edges extracted from the v-map, threshold 1 − *v* ≥ 0.1361, 4979 px (**left**); filtered KDE centroids used as SAM point prompts, 236 points, radius = 3.2 px (**right**).

The fourth step converts the continuous AT edge map into discrete, individually segmented object masks using the SAM [58]. The process involves three sub-steps: identification of candidate object centres from the edge map, generation of SAM masks via point prompts, and filtering of masks by RF probability. The AT edge map is first inverted: The boundary strength signal is defined as *b*(*x*,*y*) = 1 − *v*(*x*,*y*) so that strong edges correspond to high values. Pixels with *b* < 0.05 are considered inactive and excluded from further processing. To identify individual object locations, a spatial Kernel Density Estimation (KDE) is applied to the active edge pixels. The KDE bandwidth is set adaptively as three times the mean nearest-neighbour distance among active edge pixels, computed via a k-d tree. The resulting density map is smoothed with a Gaussian filter of the same bandwidth. Local maxima of the density map (peaks separated by at least one bandwidth radius) are taken as candidate object centres. The distribution of *b* values among active pixels is modelled by a two-component Gaussian Mixture Model. The intersection of the two fitted Gaussians is used as the threshold separating weak edges (likely noise or background texture) from strong edges (likely object boundaries). Only active pixels above this adaptive threshold are retained for KDE computation, ensuring that the density map responds to genuine structural boundaries rather than residual background signal (Figure 8).

For each candidate centre identified by the KDE, SAM is queried with a single-point prompt at the centre coordinates. SAM generates multiple candidate masks for each prompt; the mask with the highest confidence score is selected. All selected masks are kept as separate objects—no mask merging is performed at this stage, so adjacent or overlapping objects remain distinct. SAM operates on the original RGB image (not the modulated version) to preserve the full spectral contrast available for boundary delineation. The SAM used is the Vision Transformer variant B (ViT-B), which provides an optimal balance between segmentation accuracy and inference speed for large satellite images. This choice is consistent with recent benchmarking of SAM encoder variants, which shows that larger encoders generally achieve higher segmentation accuracy at the cost of processing speed and that SAM-based models can remain competitive with, though not necessarily superior to, established architectures such as Mask R-CNN, particularly on small or narrow objects [59].

**Figure 9:**
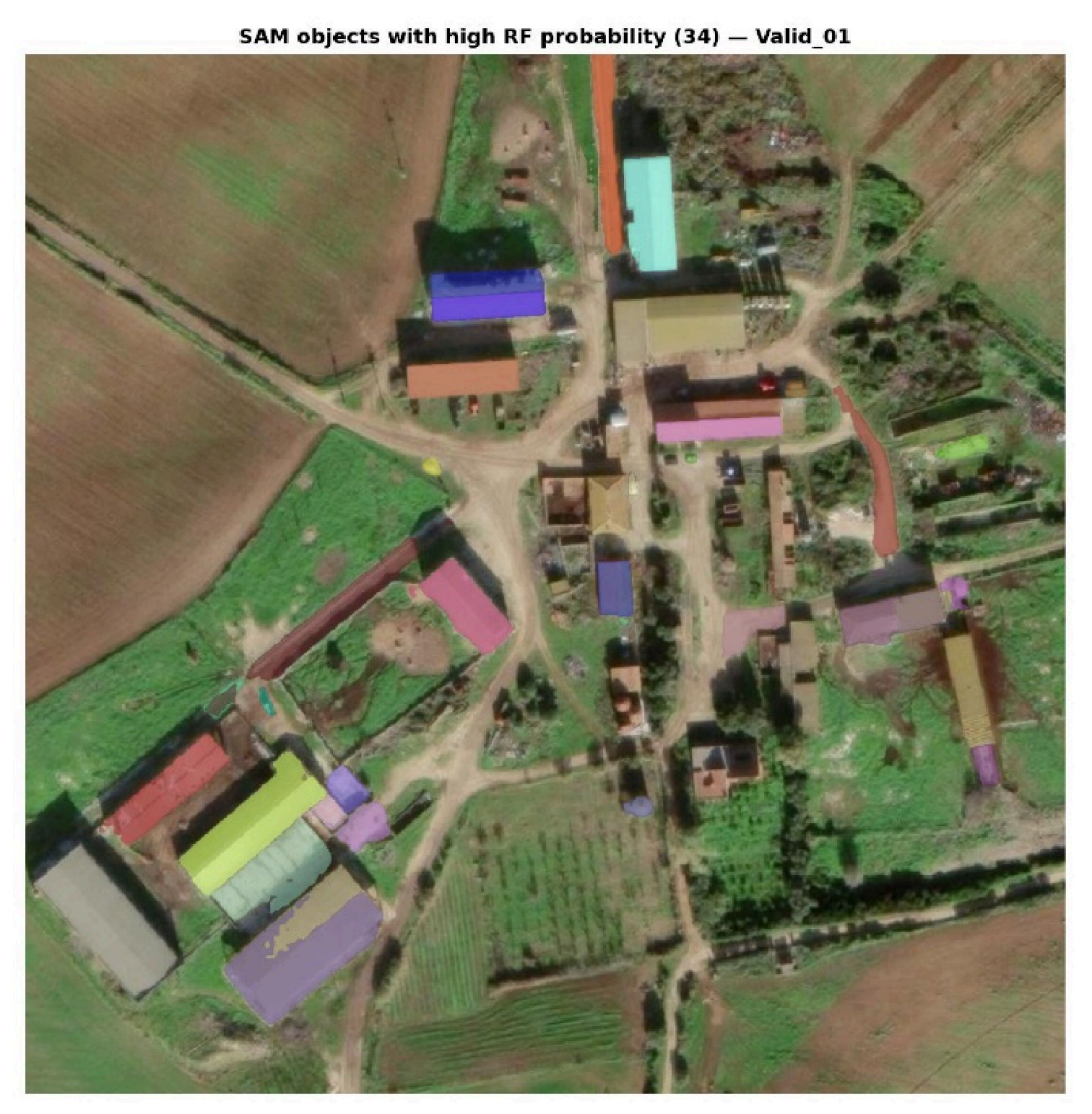
Full-pipeline output for validation scene Valid_01: 34 objects retained after SAM segmentation, geometric filtering, and non-maximum suppression.

Not all SAM-produced masks correspond to genuine infrastructure objects: Some may arise from vegetation clusters, shadows, or other high-contrast features that were not fully suppressed by the modulation step. Each mask is filtered by computing the mean RF probability over its interior pixels:

$$\bar{P_{m}}=\;\frac{1}{\left|S_{m}\right|}*\sum_{(x,y)\in S_{m}}P(x,y)$$

where *S_m_* is the set of pixels belonging to mask *m*; the distribution of $\bar{P_{m}}$ values across all masks is again modelled by a two-component GMM. Only masks in the high-probability component ($\bar{P_{m}}$ above the GMM intersection) are retained as confirmed infrastructure objects. This adaptive threshold—rather than a fixed cutoff—automatically adjusts to the difficulty and scene-specific probability range of each image. Three diagnostic images are produced at the end of Step 4: (i) *_bordi_su_rgb.jpg, showing the strong-edge pixels overlaid in red on the original RGB; (ii) *_kde_centroidi.jpg, showing the KDE density peaks with their estimated radius overlaid as cyan circles; and (iii) *_SAM_separati.jpg, showing the final filtered SAM masks in randomly assigned colours on the original RGB (Figure 9). Two additional, individually validated refinements are applied to the SAM output before the final GMM filtering step. A geometric filter based on mask solidity (the ratio of mask area to its convex-hull area) rejects non-convex, winding shapes inconsistent with compact infrastructure objects, following established practice in post-processing geometric regularisation for building extraction [60]. Separately, because a single elongated structure can generate several closely spaced KDE-derived point prompts along its length, each independently queried against SAM, a non-maximum suppression step [61] removes redundant re-detections of the same object (mutual IoU > 0.70 between candidate masks, retaining the one with the highest mean RF probability). Both refinements were verified on held-out scenes to leave recall unaffected while measurably reducing the false-positive count. All processing was carried out in Python 3.12.6 using NumPy 2.4.2, SciPy1.15.3, scikit-image 0.25.2, scikit-learn 1.6.1, and rasterio 1.5.0. SAM inference used PyTorch 2.10.0.

## 3. Experiment and Results

**Figure 10:**
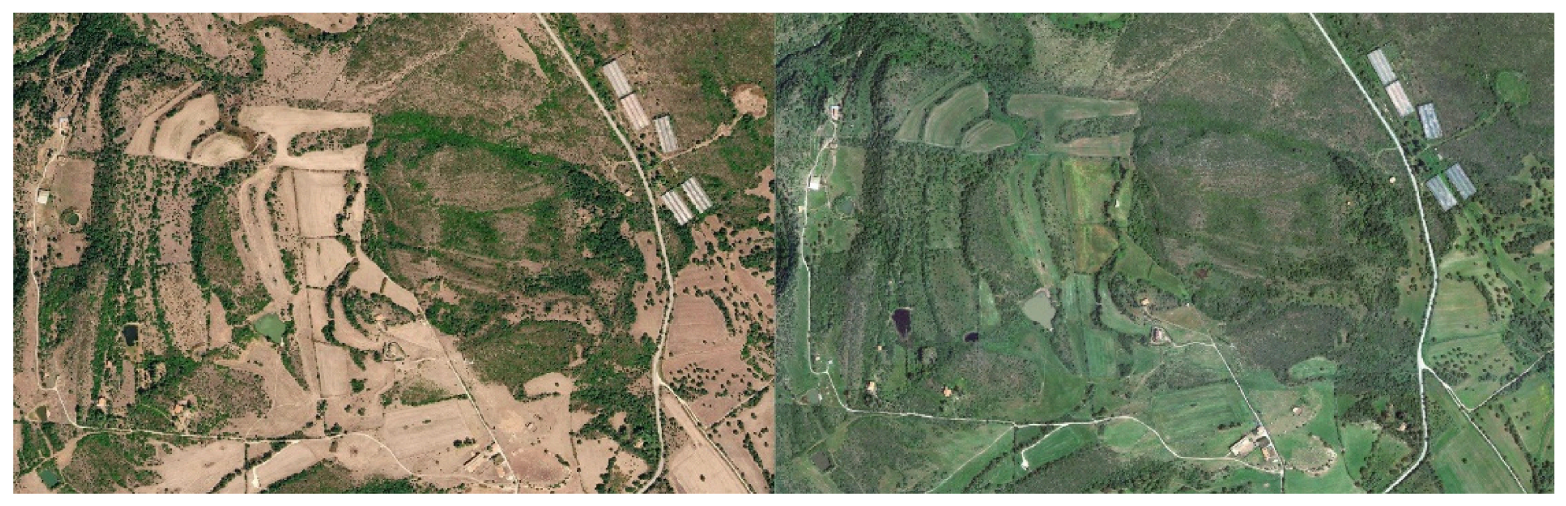
Illustrative example of the interpretive difficulty faced by classification algorithms due to the inevitable seasonal cycle.

Sardinia is the second-largest island in the Mediterranean, with an area of approximately 24,000 km^2^, and it represents one of the most strongly rural regions in the whole of Italy. This characteristic is not simply the result of a superficial reading of the territory: Several analytical models applied to the island converge in confirming its predominantly rural character, rooted in a long agro-pastoral tradition that has shaped, over the centuries, not only the economy but also the cultural and landscape identity of the region. This apparently solid picture, however, conceals worrying dynamics. Rural Sardinia is today a reality in profound transformation, caught between the crisis of the primary sector and the rise of new economies that are reshaping land use across the island: The emergence of tourism as a new economic driver is significantly altering the rural character of the island, exerting growing pressure on coastal areas, where demand for buildable land for hospitality purposes directly competes with agricultural and natural land uses [62]. A comparable rural-to-anthropogenic transition, driven by land abandonment and settlement pressure, has recently been documented for the Central Apennines in Italy through multi-temporal Landsat classification validated against CORINE Land Cover data [63]. Having tools that allow continuous and as accurate as possible monitoring of anthropogenic pressures and changes in land cover and land use is the foundation for structuring territorial planning models that are coherent with the evolution of the context. However, the challenges involved in developing such tools are far from negligible due primarily to the complexity of certain territorial contexts [64]. The intrinsic difficulty of computer vision algorithms in correctly interpreting image content stems mainly from the presence of noise and perturbations, which can lead to instability and low-quality reconstructions [65], and from the inevitable yet cyclical seasonal change affecting certain rural and environmental contexts [66] (Figure 10).

In this experimental section, we present the results obtained from the application of GANCIU. In this experimental section, we present a quantitative validation of the GANCIU pipeline on twelve validation scenes. Each validation scene was acquired from a documented and traceable source (Esri World Imagery, Vantor Vivid mosaic; sensor, acquisition date, and native resolution verified per scene via the associated metadata service) and manually annotated with instance-level ground truth: one polygon per genuine infrastructure object (building or maintained paved/unpaved road). Informal dirt trails and bare-earth clearings were deliberately excluded, as they do not constitute infrastructure under the definition adopted in this study.

Predicted and ground-truth objects were matched using the Hungarian algorithm [67] applied to a pairwise Intersection-over-Union (IoU) cost matrix, with a match accepted as a true positive at IoU ≥ 0.5, following standard practice in instance-segmentation evaluation [68]. For each scene, we computed precision, recall, F1 score, and mean IoU over matched objects; aggregate statistics (mean ± standard deviation) are reported over the twelve scenes. To quantify the contribution of the modulation-AT-SAM-GMM stages beyond the Random Forest classifier alone, we additionally evaluated an RF-only baseline: the same RF probability map, thresholded via the identical adaptive Otsu procedure described in Section 2.1 and labelled via connected components, with no modulation, edge detection, SAM segmentation, or GMM filtering applied (Table 4).

**Figure 11:**
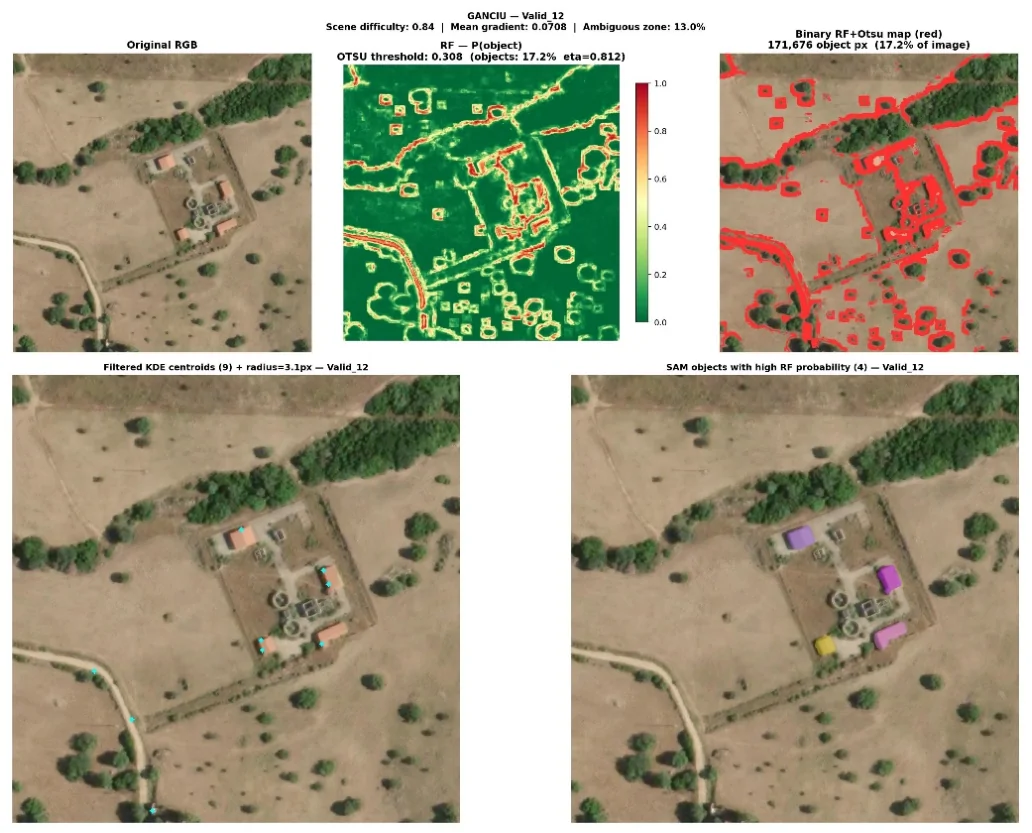
Full pipeline output for validation scene Valid_12: original RGB (**top-left**); RF probability map with adaptive Otsu threshold = 0.308, η = 0.812 (**top-middle**); binary RF + Otsu map, 171,676 object pixels—17.2% of the image (**top-right**); filtered KDE centroids, 9 points, radius = 3.1 px (**bottom-left**); final objects after SAM segmentation and filtering, 4 objects (**bottom-right**).

The RF-only baseline is essentially non-functional as an object detector: Pooled across the twelve scenes, it yields a precision of 0.001 (8320 false positives against only five true positives) and a recall of 0.036 (mean 0.036 ± 0.063, F1 0.004 ± 0.002, mean IoU on the rare, matched objects 0.666 ± 0.126). The full pipeline reduces pooled false positives by close to two orders of magnitude (209 versus 8320) while increasing true positives nearly twentyfold (95 versus five), confirming that the modulation-AT-SAM-GMM sequence contributes substantially beyond the per-pixel classifier alone rather than merely re-expressing its decision boundary. Performance varies considerably across scenes, reflecting genuine differences in scene complexity rather than a systematic bias. Valid_12, an isolated compound in open, unobstructed terrain, achieves perfect precision (1.000) with zero false positives (Figure 11).

**Figure 12:**
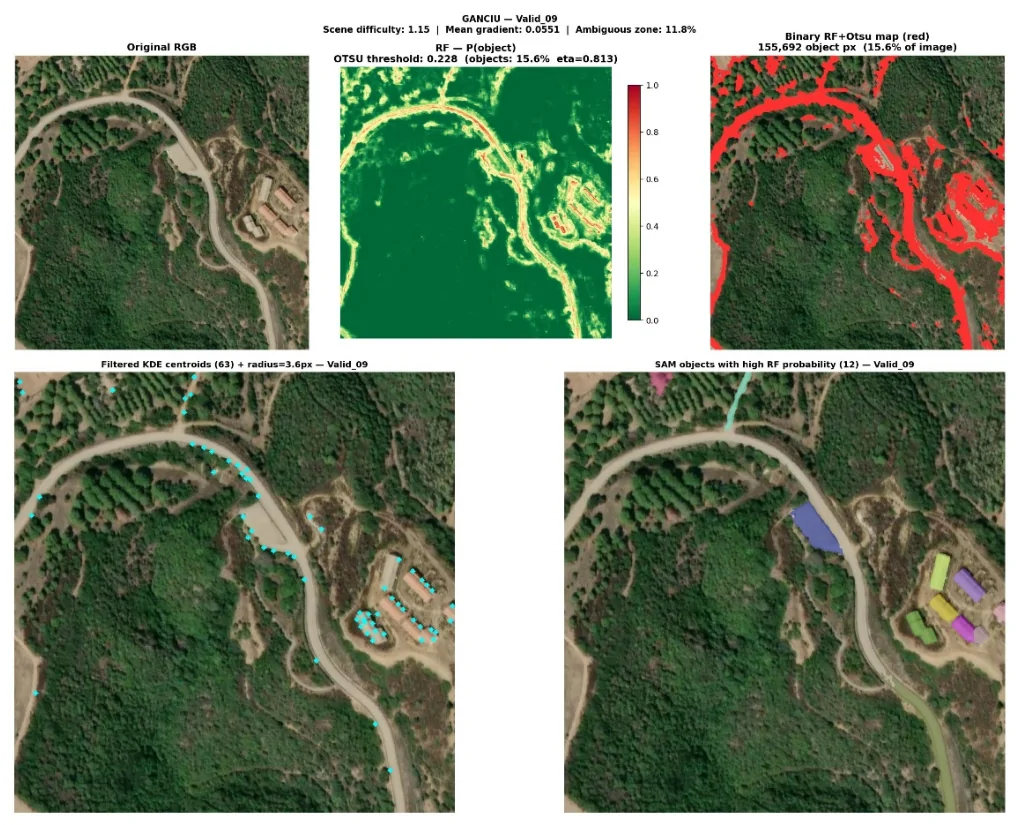
Full pipeline output for validation scene Valid_09: original RGB (**top-left**); RF probability map with adaptive Otsu threshold = 0.228, η = 0.813 (**top-middle**); binary RF + Otsu map, 155,692 object pixels—15.6% of the image (**top-right**); filtered KDE centroids, 63 points, radius = 3.6 px (**bottom-left**); final objects after SAM segmentation and filtering, 12 objects (**bottom-right**).

Valid_09 (Figure 12) achieves the highest F1 score (0.818). Mean IoU on matched objects is both high and remarkably stable across scenes (0.742 ± 0.060), indicating that when the pipeline correctly identifies an object, it delineates its boundary with consistent geometric accuracy regardless of overall scene difficulty.

**Figure 13:**
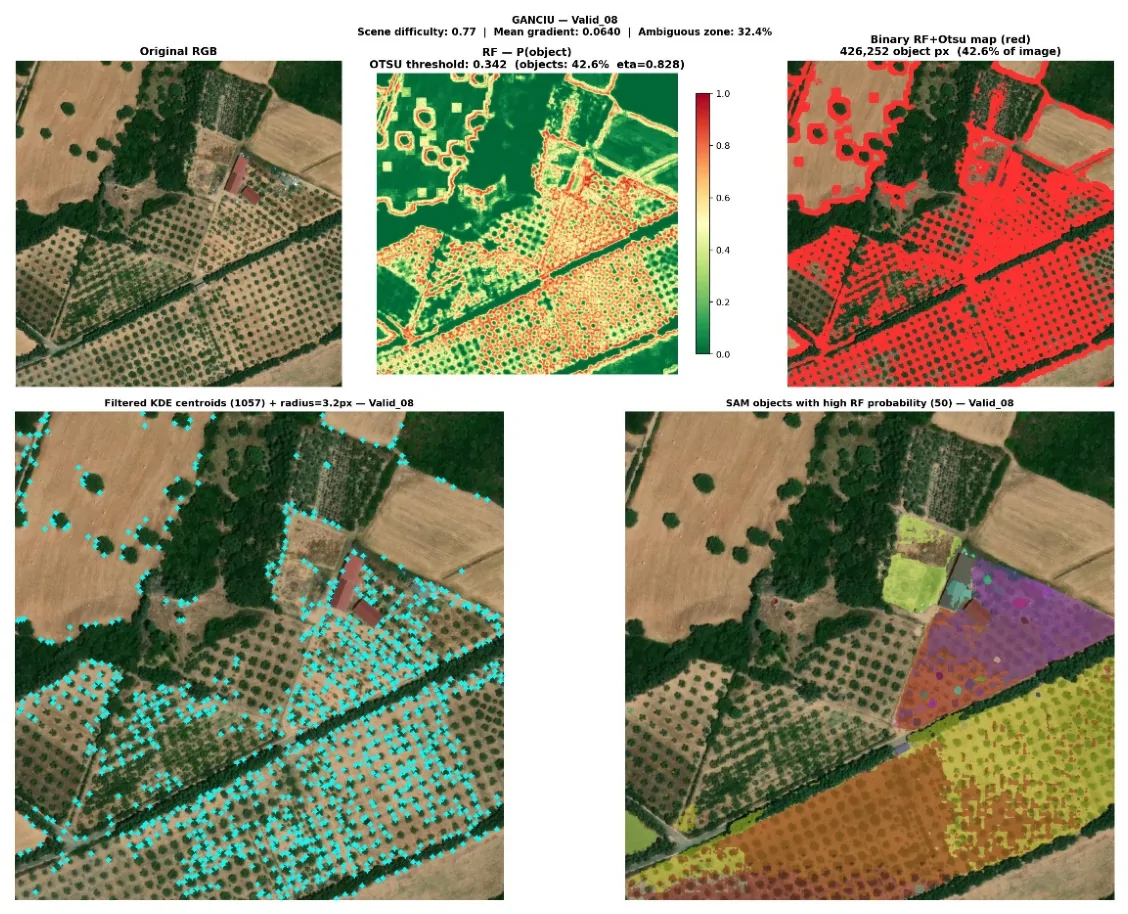
Full pipeline output for validation scene Valid_08: original RGB (**top-left**); RF probability map with adaptive Otsu threshold = 0.342, η = 0.828 (**top-middle**); binary RF + Otsu map, 426,252 object pixels—42.6% of the image (**top-right**); filtered KDE centroids, 1057 points, radius = 3.2 px (**bottom-left**); final objects after SAM segmentation and filtering, 50 objects (**bottom-right**).

Visual inspection of the per-scene outputs reveals three largely distinct sources of residual false positives rather than a single undifferentiated failure mode. First, informal dirt trails winding across open terrain generate elongated, low-solidity SAM masks; a dedicated geometric filter (Section 2.4) removes many of these at no measurable cost to recall, though it cannot, by construction, distinguish a winding informal trail from a winding but legitimate unpaved road, since both share the same non-convex shape signature. Second, straight road or watercourse segments—which a solidity-based filter must retain to avoid discarding legitimately elongated structures such as long warehouses—remain as false positives when they do not correspond to an annotated object. Third and most prominent in the lowest-precision scene (Valid_08, precision = 0.080, 46 false positives out of 50 predictions), regularly spaced rows of orchard or olive-grove trees produce a periodic spatial texture that plausibly triggers the Gabor-derived features in a manner similar to genuine structural regularity; consistent with this reading, Valid_08 also has the highest Random Forest object-coverage percentage among the twelve scenes (Figure 13).

**Figure 14:**
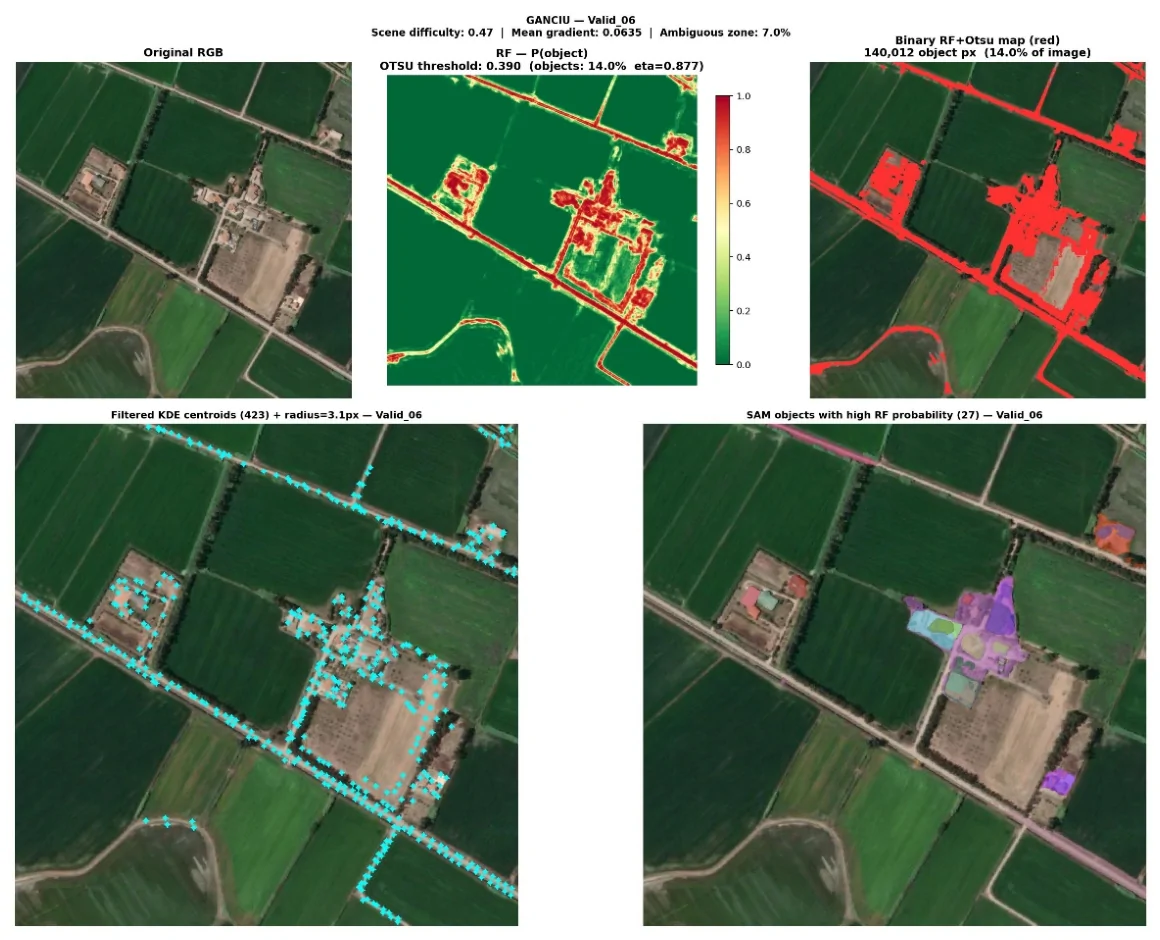
Full pipeline output for validation scene Valid_06: original RGB (**top-left**); RF probability map with adaptive Otsu threshold = 0.390, η = 0.877 (**top-middle**); binary RF + Otsu map, 140,012 object pixels—14.0% of the image (**top-right**); filtered KDE centroids, 423 points, radius = 3.1 px (**bottom-left**); final objects after SAM segmentation and filtering, 27 objects (**bottom-right**).

A fourth, distinct phenomenon affects matched-object quality rather than the false-positive count: in scenes with tightly clustered adjacent buildings (Valid_06, mean IoU = 0.631, the lowest of the twelve scenes), adjacent structures are occasionally merged within a single SAM mask, reducing localisation precision without necessarily causing a missed detection (Figure 14).

## 4. Discussion

The quantitative validation reported in Section 3 provides the first rigorous evidence for the contribution of each stage of the GANCIU pipeline beyond the per-pixel Random Forest classifier alone. The comparison with the RF-only baseline is the most direct indicator of this: Pooled across twelve validation scenes, the full pipeline reduces false positives from 8320 to 209, close to two orders of magnitude, while increasing true positives from 5 to 95, nearly twentyfold. Because both figures are computed from the identical RF probability map, this difference cannot be attributed to a better classifier; it reflects the combined effect of RF-guided modulation, Ambrosio–Tortorelli edge detection, SAM-based delineation, and GMM filtering acting on that map. This addresses, empirically, a concern that could otherwise be raised about the pipeline’s internal logic: Since both the modulation step and the final GMM filter are derived from the same RF probability signal, one might question whether the intermediate stages contribute independent evidence or simply re-express RF’s own decision boundary in a different form. The RF-only baseline shows that they do not merely re-express it as a simple threshold, and a label of the same probability map performs catastrophically worse, confirming that SAM’s segmentation (computed on the original RGB image using a visual representation entirely independent of the RF/GLCM/Gabor feature space) and the subsequent geometric filtering introduce genuine additional constraints rather than redundant computation. Mean IoU on correctly matched objects (0.742 ± 0.060) is both high and, notably, far more stable across scenes than precision or recall (which range from 0.080 to 1.000 and from 0.429 to 1.000, respectively). This pattern indicates that scene difficulty affects primarily the pipeline’s ability to correctly identify which regions are genuine infrastructure and not its ability to delineate an object’s boundary once identified. In other words, GANCIU’s failure mode is predominantly one of detection completeness and purity rather than of localisation accuracy—a distinction with direct implications for where future development effort is best directed (see below). Visual inspection of the twelve validation outputs indicates that residual false positives do not arise from one undifferentiated weakness but from at least three largely distinct mechanisms. The first is confusion with informal, winding dirt trails, for which their spectral and textural similarity to bare or lightly vegetated ground makes them difficult for a texture-based classifier to reject; the geometric filter introduced in Section 2.4 (rejecting low-solidity, non-convex masks) removes many of these at no measurable cost to recall, though it cannot, by construction, distinguish a winding informal trail from a winding but legitimate unpaved road, since both present the same non-convex shape signature. The second mechanism is straight linear features—road or watercourse segments without ground truth correspondence, which the same geometric filter cannot address, since it must retain straight elongated shapes to avoid discarding legitimate long structures such as warehouses; this is an intrinsic trade-off of a shape-only criterion, not a defect of its implementation. The third and most consequential in the lowest-precision scene (Valid_08, precision = 0.080, 46 false positives out of 50 predictions) is a periodic spatial texture produced by regularly spaced orchard or olive-grove rows, which plausibly interacts with the Gabor-derived features explicitly designed to respond to oriented periodic patterns in a manner similar to genuine structural regularity; consistent with this interpretation, Valid_08 also has the highest RF object-coverage percentage among the twelve scenes. This hypothesis is plausible and consistent with the available evidence but was not tested by ablating the Gabor features directly, and we note this as a specific, falsifiable direction for future verification rather than an established mechanism. A fourth, qualitatively different phenomenon affects match quality rather than the false-positive count: in scenes with tightly clustered adjacent buildings (Valid_06, mean IoU = 0.631, the lowest of the twelve scenes), SAM occasionally merges adjacent structures into a single mask, reducing localisation precision without necessarily causing a missed detection. The two post-processing refinements introduced in this revision, the geometric solidity filter and non-maximum suppression for duplicate redetections, were each verified independently on held-out data to leave recall unaffected while measurably reducing false positives, rather than being adopted on the basis of aggregate metrics alone. This distinction matters methodologically: An aggregate precision gain could, in principle, mask a harmful trade-off in a subset of scenes, and confirming a zero-recall-cost outcome at the level of individual scenes is a stronger form of evidence than an improved mean. The NMS step, in particular, addresses a failure mode that is easy to overlook when only inspecting aggregate counts: A single elongated structure can generate several closely spaced point prompts along its length, each independently re-detecting the same object via SAM so that a naïve one-to-one instance matching evaluation would penalise redundant-but-correct detections as if they were errors. Several limitations should be stated explicitly. First, the RF classifier relies exclusively on RGB-derived texture and colour descriptors without near-infrared bands or vegetation indices such as NDVI that are commonly used elsewhere in the remote-sensing literature specifically to separate vegetated from bare or impervious surfaces; this constraint plausibly contributes to the confusion between infrastructure and the bare or sparsely vegetated ground discussed above, and its removal is a natural target for future work rather than a fundamental barrier to the approach. Second, the modulation transition parameter γ was held fixed at 1.0 throughout this validation. While the other parameters of the pipeline (the Otsu threshold, the reliability metric η, the AT regularisation weight μ, and the ε-annealing schedule) were confirmed to vary meaningfully and appropriately across the twelve scenes (η ranged from 0.776 to 0.877, μ from 1.27 to 4.97, and the ε schedule selector chose the medium-length schedule for the single lowest-difficulty scene rather than the long schedule used elsewhere), γ was not calibrated adaptively, and doing so is identified as a specific, currently unimplemented direction for improvement. Third, the pipeline’s architecture is a forward, progressively conditioned chain rather than one incorporating true feedback: Later stages constrain but do not correct earlier ones, so an error introduced at the RF stage cannot currently be revised in light of downstream evidence from AT or SAM. Fourth, although the twelve validation scenes span multiple acquisition dates and, in several cases, different sensors (WorldView-3 and the WorldView Legion constellation), offering partial evidence of robustness to acquisition conditions, no systematic, controlled study isolating individual factors such as illumination, season, or sensor was carried out, and the geographic scope remains limited to rural Sardinia. Taken together, these results position GANCIU as an interpretable, training-data-efficient alternative to end-to-end deep-learning segmentation for infrastructure extraction; each stage remains individually inspectable, and its failure modes are individually attributable, in contrast to a monolithic network for which its errors are comparatively difficult to localise to a specific mechanism while also making its current limitations concrete and falsifiable rather than implicit. The three largely independent false-positive mechanisms identified above, together with the RGB-only spectral constraint, motivate the targeted developments outlined in the following section.

## 5. Conclusions

This study introduced GANCIU, a hybrid pipeline combining a per-pixel Random Forest classifier, RF-probability-guided image modulation, Ambrosio–Tortorelli edge detection, and Segment Anything Model-based delineation for the extraction of man-made infrastructure from very-high-resolution satellite imagery. Beyond the qualitative characterisation across fourteen scenes in Sardinia (Section 3), this revision added a rigorous quantitative validation on twelve independent, geographically disjoint scenes with instance-level ground truth, demonstrating that the full pipeline reduces false positives by close to two orders of magnitude relative to a Random-Forest-only baseline (209 versus 8320 pooled false positives) while increasing true positives nearly twentyfold, with a mean IoU of 0.742 ± 0.060 on correctly matched objects. Two individually validated post-processing refinements, a geometric shape filter and a duplicate-detection step, further improved precision at no measurable cost to recall. Residual false positives were traced to three largely distinct mechanisms: informal winding trails, straight linear features without ground-truth correspondence, and periodic orchard or olive-grove textures, each suggesting a specific, targeted direction for future refinement rather than a single undifferentiated weakness. Taken together, these results support GANCIU as a viable, interpretable alternative to end-to-end deep learning segmentation for infrastructure extraction in complex rural landscapes while transparently documenting its current limitations: reliance on RGB-only spectral information without near-infrared bands or vegetation indices, a fixed modulation transition parameter, and residual sensitivity to periodic agricultural textures. These limitations provide concrete, falsifiable targets for the future developments outlined above. To improve the overall precision of GANCIU, future developments are directed along three converging lines. First, the training dataset will be enriched through targeted sampling of intensive agricultural surfaces—including vineyard rows, olive groves, and greenhouse structures—supported by data augmentation techniques, with the aim of increasing the representation of spectrally ambiguous backgrounds without a proportional increase in annotation effort. Second, geometric constraints based on mask compactness and aspect ratios will be introduced in the final filtering stage, exploiting the known morphological regularity of buildings and roads to distinguish them from the elongated and periodic patterns of row crops. Third, the final filter will be reformulated to combine RF probability with shape descriptors derived from SAM, replacing the current single-criterion GMM threshold with a multi-feature decision boundary capable of separating genuine infrastructure masks from agricultural false positives even when their mean internal RF probability overlaps substantially. A further, complementary direction would be the introduction of an explicit feedback mechanism, analogous to reinforcement-learning-based iterative refinement recently proposed for remote sensing change detection, allowing later stages of GANCIU to inform and correct the earlier RF classification rather than only propagating its output downstream [69]. Of the three lines above, the second (geometric constraints based on mask shape) has since been implemented and validated, as described in Section 2.4 and evaluated quantitatively in Section 3; the first (targeted training-set enrichment) and third (a combined RF-probability/shape decision boundary) remain directions for future work, alongside the feedback mechanism described above.

## Figures and Tables

**Table 1:** A classification of algorithms according to supervised versus unsupervised and parametric versus non-parametric methodologies.

	Parametric	Non-parametric
**Supervised**	Maximum Likelihood Classifier (MLC); Logistic Regression	Random Forest (RF) Support Vector Machine (SVM) K-Nearest Neighbours (KNNs) Neural Networks (ANNs)
**Unsupervised**	Gaussian Mixture Models (GMMs)	K-Means Iterative Self-Organizing Data Analysis Technique (ISODATA) Density-Based Spatial Clustering of Applications with Noise (DBSCAN) Hierarchical Clustering

**Table 2:** Sensor, acquisition date, scene type, and bounding-box coordinates (EPSG:23032, ED50/UTM zone 32N) for the 44 scenes used to train the Random Forest classifier.

Image_ID	Sensor	Date	Scene Type	*Xmin*	*Xmax*	*Ymin*	*Ymax*
Train_01	WorldView-3	18 April 2025	Rural Farm	435,746	436,261	4,522,265	4,522,527
Train_02	WorldView-3	10 December 2023	Rural Farm	448,850	449,250	4,508,915	4,509,200
Train_03	WorldView-3	15 September 2025	Rural Farm	517,221	517,605	4,513,565	4,513,839
Train_04	WorldView-3	2 August 2025	Rural Farm	542,049	542,464	4,523,687	4,523,982
Train_05	WorldView Legion-1	10 April 2025	Rural Farm	469,975	470,381	4,387,770	4,388,060
Train_06	WorldView-3	26 June 2025	Rural Farm	470,103	470,445	4,350,183	4,350,426
Train_07	WorldView Legion-1	25 June 2025	Rural Farm	454,054	454,396	4,342,886	4,343,129
Train_08	WorldView-3	26 June 2025	Rural Farm	467,901	468,266	4,320,329	4,320,590
Train_09	WorldView-3	14 November 2023	Rural Farm	497,904	498,266	4,322,289	4,322,547
Train_10	WorldView-3	7 June 2025	Rural Farm	549,001	549,362	4,400,909	4,401,166
Train_11	WorldView Legion-1	30 April 2025	Rural Farm	521,958	522,392	4,427,579	4,427,888
Train_12	WorldView-3	19 May 2025	Rural Farm	515,904	516,318	4,436,149	4,436,444
Train_13	WorldView Legion-2	2 May 2025	Rural Village	509,844	510,202	4,439,235	4,439,490
Train_14	GeoEye-1	17 September 2023	Rural Village	539,924	540,374	4,506,062	4,506,382
Train_15	WorldView-3	14 June 2025	Rural Village	546,609	547,009	4,514,434	4,514,719
Train_16	WorldView-3	2 August 2025	Rural Village	548,073	548,492	4,515,885	4,516,184
Train_17	WorldView-3	26 June 2025	Rural Village	474,975	475,315	4,321,014	4,321,257
Train_18	WorldView-3	26 June 2025	Rural Village	462,322	462,720	4,361,084	4,361,367
Train_19	WorldView Legion-1	10 April 2025	Rural Village	469,593	470,011	4,420,505	4,420,802
Train_20	WorldView-3	14 October 2023	Rural Village	439,280	439,662	4,502,063	4,502,335
Train_21	WorldView Legion-2	28 June 2025	Rural Village	518,401	518,783	4,551,058	4,551,329
Train_22	WorldView Legion-2	28 June 2025	Farmland and roads	518,128	518,529	4,551,818	4,552,104
Train_23	WorldView-3	18 April 2025	Farmland and roads	444,122	444,543	4,505,893	4,506,193
Train_24	WorldView Legion-1	6 June 2025	Farmland and roads	480,445	480,827	4,387,794	4,388,066
Train_25	WorldView-3	14 November 2023	Farmland and roads	498,869	499,251	4,323,518	4,323,790
Train_26	WorldView Legion-6	18 August 2025	Farmland and roads	546,257	546,639	4,346,828	4,347,100
Train_27	WorldView Legion-2	28 May 2025	Farmland and roads	526,039	526,420	4,445,841	4,446,113
Train_28	WorldView-3	18 April 2025	Agricultural area only	443,650	444,060	4,509,462	4,509,753
Train_29	WorldView-3	10 December 2023	Agricultural area only	448,589	448,929	4,510,528	4,510,771
Train_30	WorldView Legion-1	2 July 2025	Agricultural area only	535,855	536,195	4,532,741	4,532,983
Train_31	WorldView Legion-1	2 July 2025	Agricultural area only	534,227	534,544	4,535,987	4,536,213
Train_32	WorldView Legion-3	18 July 2025	Agricultural area only	463,130	463,416	4,424,792	4,424,995
Train_33	WorldView Legion-3	18 July 2025	Agricultural area only	462,084	462,370	4,424,658	4,424,862
Train_34	WorldView-3	26 June 2025	Agricultural area only	466,581	466,866	4,346,190	4,346,394
Train_35	WorldView-3	26 June 2025	Agricultural area only	463,205	463,505	4,346,272	4,346,485
Train_36	WorldView-3	14 November 2023	Agricultural area only	498,977	499,277	4,322,576	4,322,790
Train_37	WorldView-3	7 June 2025	Agricultural area only	550,027	550,341	4,388,094	4,388,319
Train_38	WorldView-3	7 June 2025	Agricultural area only	549,278	549,593	4,397,994	4,398,219
Train_39	WorldView-3	7 June 2025	Agricultural area only	549,122	549,453	4,398,608	4,398,844
Train_40	WorldView-3	7 June 2025	Agricultural area only	553,732	554,079	4,407,624	4,407,872
Train_41	WorldView-3	14 October 2023	Agricultural area only	442,185	442,454	4,4951,27	4,495,319
Train_42	WorldView-3	14 October 2023	Agricultural area only	441,375	441,645	4,494,237	4,494,429
Train_43	WorldView-3	26 June 2025	Agricultural area only	482,938	483,300	4,345,204	4,345,463
Train_44	WorldView Legion-1	25 June 2025	Agricultural area only	458,577	458,940	4,327,581	4,327,840

**Table 3:** Summary of Random Forest training: dataset composition, training time, out-of-bag score, and test-set classification performance.

Parameter	Value
Raw samples extracted	21,503,381
Samples used after balancing	17,850,000
*Object samples*	916,662 (5.1%)
*Background samples*	16,933,338 (94.9%)
Number of trees	300
Training time	28,829 s (≈8.0 h)
OOB score	0.963
Test set accuracy	0.963
Background (precision/recall/F1)	0.999/0.962/0.980
Object (precision/recall/F1)	0.581/0.980/0.730

**Table 4:** Sensor, acquisition date, scene type, and bounding-box coordinates (EPSG:23032, ED50/UTM zone 32N) for the 12 independent scenes used for quantitative validation.

Scene	GT obj	Pred	TP	FP	FN	Precision	Recall	F1	MeanIoU
Valid_01	18	34	8	26	10	0.235	0.444	0.308	0.755
Valid_02	7	17	3	14	4	0.176	0.429	0.250	0.856
Valid_03	2	7	2	5	0	0.286	1.000	0.444	0.769
Valid_04	4	8	3	5	1	0.375	0.750	0.500	0.786
Valid_05	15	29	12	17	3	0.414	0.800	0.545	0.779
Valid_06	18	27	11	16	7	0.407	0.611	0.489	0.631
Valid_07	21	33	15	18	6	0.455	0.714	0.556	0.779
Valid_08	5	50	4	46	1	0.080	0.800	0.145	0.681
Valid_09	10	12	9	3	1	0.750	0.900	0.818	0.740
Valid_10	18	34	11	23	7	0.324	0.611	0.423	0.693
Valid_11	19	49	13	36	6	0.265	0.684	0.382	0.693
Valid_12	5	4	4	0	1	1.000	0.800	0.889	0.743
Mean ± SD						0.397 ± 0.253	0.712 ± 0.169	0.479 ± 0.213	0.742 ± 0.060

## Data Availability

The original contributions presented in this study are included in the article. Further inquiries can be directed to the corresponding author.
